# Recent advances in understanding the reinforcing ability and mechanism of carbon nanotubes in ceramic matrix composites

**DOI:** 10.1088/1468-6996/15/6/064902

**Published:** 2014-12-29

**Authors:** Mehdi Estili, Yoshio Sakka

**Affiliations:** 1 International Center for Young Scientists (ICYS), National Institute for Materials Science (NIMS), 1-2-1 Sengen, Tsukuba 305-0047, Japan; 2 Advanced Ceramics Group, Materials Processing Unit, National Institute for Materials Science (NIMS), 1-2-1 Sengen, Tsukuba 305-0047, Japan

**Keywords:** carbon nanotubes, ceramic matrix composites, mechanical properties

## Abstract

Since the discovery of carbon nanotubes (CNTs), commonly referred to as ultimate reinforcement, the main purpose for fabricating CNT–ceramic matrix composites has been mainly to improve the fracture toughness and strength of the ceramic matrix materials. However, there have been many studies reporting marginal improvements or even the degradation of mechanical properties. On the other hand, those studies claiming noticeable toughening measured using indentation, which is an indirect/unreliable characterization method, have not demonstrated the responsible mechanisms applicable to the nanoscale, flexible CNTs; instead, those studies proposed those classical methods applicable to microscale fiber/whisker reinforced ceramics without showing any convincing evidence of load transfer to the CNTs. Therefore, the ability of CNTs to directly improve the macroscopic mechanical properties of structural ceramics has been strongly questioned and debated in the last ten years. In order to properly discuss the reinforcing ability (and possible mechanisms) of CNTs in a ceramic host material, there are three fundamental questions to our knowledge at both the nanoscale and macroscale levels that need to be addressed: (1) does the intrinsic load-bearing ability of CNTs change when embedded in a ceramic host matrix?; (2) when there is an intimate atomic-level interface without any chemical reaction with the matrix, could one expect any load transfer to the CNTs along with effective load bearing by them during crack propagation?; and (3) considering their nanometer-scale dimensions, flexibility and radial softness, are the CNTs able to improve the mechanical properties of the host ceramic matrix at the macroscale when individually, intimately and uniformly dispersed? If so, how? Also, what is the effect of CNT concentration in such a defect-free composite system? Here, we briefly review the recent studies addressing the above fundamental questions. In particular, we discuss the new reinforcing mechanism at the nanoscale responsible for unprecedented, simultaneous mechanical improvements and highlight the scalable processing method enabling the fabrication of defect-free CNT-concentered ceramics and CNT-graded composites with unprecedented properties. Finally, possible future directions will be briefly presented.

## 1. Introduction

Carbon nanotubes (CNTs), with their remarkable axial strength and stiffness, chemical/thermal stability and outstanding flexibility, near-perfect, one-dimensional crystalline structure and high aspect ratio, have been considered, in theory, as an ultimate additive to improve the macroscopic mechanical properties of structural ceramics such as Al_2_O_3_, Si_3_N_4_ and ZrO_2_ [1–16]. In practice, however, there have been many studies reporting marginal improvements or even the degradation of mechanical properties after the CNT addition [15–43]. Perhaps, common well-known issues, such as the agglomeration of CNTs, a poor CNT–ceramic interface and CNT damage during treatment/sintering processes, could be the reasons for such disappointing mechanical properties, preventing the reinforcing ability of the CNTs from being exploited [1, 44]. In addition, in the studies claiming noticeable toughening measured using indentation, which is widely accepted as an indirect/unreliable toughness measurement method [18, 45], it has not been clear whether or not the load was transferred to the CNTs and effectively borne by them during the crack propagation. As a result, the reinforcing ability of the CNTs in a ceramic host matrix has been strongly questioned and debated in the last ten years [1].

In order to properly discuss and understand the reinforcing ability and mechanism of CNTs in a ceramic material, there are three vital questions that needed to be addressed to our knowledge at both the nanoscale and macroscale: (1) does the intrinsic load bearing ability of CNTs change when embedded in a ceramic matrix, considering the high temperature/pressure generally applied during the fabrication process and the residual misfit stresses?; (2) when there is an intimate atomic-level interface without any chemical reaction with the matrix, could one expect any load transfer to the CNTs, along with an effective load bearing by them during crack propagation?; and (3) considering their nanometer-scale dimension, flexibility and radial softness, are the CNTs able to improve the mechanical properties of the host ceramic matrix at the macroscale when individually, intimately and uniformly dispersed within the pore-free and structurally uniform ceramic matrix? If so, how? Also, what is the effect of CNT concentration in such a defect-free composite system, especially at large concentrations? Answering these questions indeed demands a simple, defect-free and structurally uniform composite system in which the individually dispersed CNTs at various concentrations are uniformly distributed and intimately embedded within the ceramic matrix with atomic level, pore-free and physical interfaces. We refer hereafter to such a system as a ‘clean’ composite system.

Here, we review the recent works studying the direct reinforcing ability (and possible mechanisms) of CNTs in a ceramic material and address the above vital questions. These studies mainly involved: (1) scalable fabrication of defect-free multi-walled CNT (MWCNT)–Al_2_O_3_ matrix composites in which individual MWCNTs with different concentrations (up to 20 vol %) were uniformly dispersed and intimately embedded within the nanostructured, pore-free and structurally uniform Al_2_O_3_ matrix (such ‘clean’ composites were essential for such investigations [17, 44, 46]); (2) direct *in situ* characterization of the interfacial load transfer and the load bearing ability of individual MWCNTs when embedded in the Al_2_O_3_ matrix [9, 10, 47]; and (3) the characterization of macroscopic mechanical properties of such fully dense, defect-free and structurally uniform composites with different MWCNT loadings [1]. In particular, we discuss the new reinforcing mechanism at the nanoscale, referred to as ‘highly energy-dissipating multiwall-type failures and plastic buckling,’ which is responsible for unprecedented, simultaneous strengthening, toughening and softening of the matrix [1]. Furthermore, we highlight the scalable processing method enabling the fabrication of a defect-free functionally graded CNT–Al_2_O_3_ composite [48, 49], as well as the most CNT-concentered ceramic bulk ever (20 vol %), with nearly theoretical density, showing superb, record-breaking electrical conductivity (∼5000 S m^−1^ at room temperature), doubled strain tolerance and perfect high-temperature compressive plasticity [1, 44, 50]. Finally, possible future directions will be briefly presented.

## 2. Scalable processing

As mentioned in the introduction, in order to properly investigate the reinforcing ability and possible reinforcing mechanisms of CNTs in a ceramic matrix, at first, a simple, fully dense, individually/uniformly dispersed CNT–ceramic composite system, which is effectively free of pores, CNTs agglomeration or damage, porous interfaces and grain-size non-uniformity, is strongly required. Such a simple system is an ideal platform for an effective load or for charge carrying by the CNTs, if any. However, the facile agglomeration of the hydrophobic pristine CNTs due to their large specific surface area and thus strong attractive van der Waals forces, their poor compatibility with ceramic materials and their damage during the initial chemical treatment and/or high-temperature sintering process are serious obstacles for the fabrication of such fully dense, high-quality CNT–ceramic composites in which the individual, crystalline CNTs—in a wide range of concentrations—are uniformly and intimately dispersed within the matrix in three dimensions over centimeter-scale distances [15–17, 44, 46]. In fact, the structural uniformity and porosity of the ceramic matrix after densification, which in turn dictate the macroscopic structural and functional performance of the composite, are strongly sensitive to the compatibility, uniformity and agglomeration level of the CNT network. To effectively address these challenges, especially at a large-scale enabling mass-production level attractive for the industry and for real applications, there would evidently be less room for the conventional methods proposed earlier. These mainly include the simple wet mixing of CNT agglomerates and ceramic powders [18, 30, 37, 38]; beads milling using small 15–50 *μ*m beads [41]; jet milling using a diamond nozzle by high-pressure pump [39, 40]; *in situ* growth of ceramic clusters on poorly dispersed CNTs in a solution that necessarily has high ionic strength and poor colloidal stability [25]; solid-state, *in situ*, metal catalyst-assisted chemical vapor deposition (CVD) synthesis of low-quality, hydrophobic CNTs within a metal-ceramic powder framework [26]; and the conventional hetero-coagulation method [27]. In the wet methods, even if the CNTs are initially dispersed in a particular solvent through well-known surface functionalization routes followed by sonication [17, 51, 52], they would severely re-agglomerate upon solvent removal or the addition of ceramic-source precursor ions, reducing the zeta potential and colloidal stability. Also, the *in situ* CVD-assisted synthesis could lead to the poor CNTs’ crystallinity, purity or surface compatibility and porous interfaces. These methods, even if shown to be successful at a small scale, are apparently not scalable and are unable to increase the CNT concentration in the matrix beyond ∼5 vol % without sacrificing the mechanical properties.

**Figure 1.**
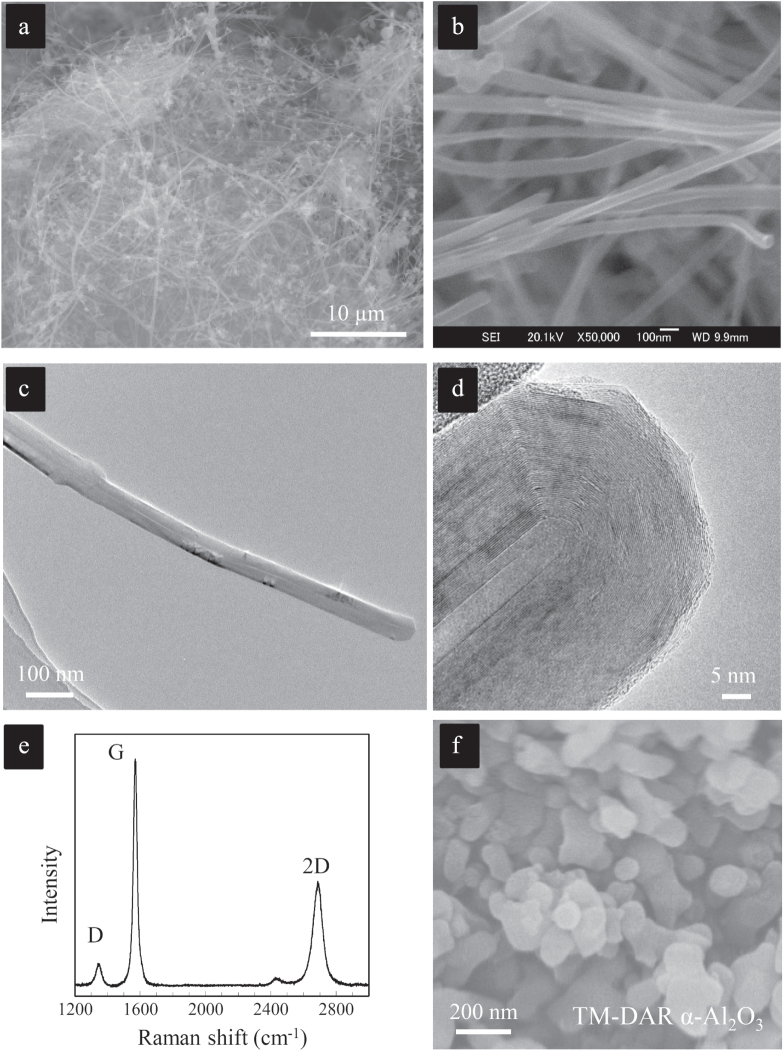
High-quality starting materials used to fabricate the ‘clean’ CNT–ceramic composite system. (a), (b) SEM images; (c), (d) TEM images; and (e) Raman spectrum of the commercially available, catalytic CVD-grown, graphitized, pristine MWCNTs characterized by long and linear macro-morphology, a high aspect ratio exceeding 100, negligible metallic impurities (<450 ppm), highly straight and crystalline walls, small inner diameters and outer diameters in the range of 20–70 nm. (f) SEM image of the ceramic particles, which are composed of a commercially available, high quality TM-DAR grade *α*-Al_2_O_3_ powder with 150 nm average particle size [9, 17]. Reprinted from M Estili and A Kawasaki 2010 *Adv. Mater*. **22** 607 (Copyright © 2010 WILEY-VCH Verlag GmbH & Co. KGaA, Weinheim) and M Estili *et al* 2008 *Acta Mater*. **56** 4070 (Copyright 2008 with permission from Elsevier).

**Figure 2.**
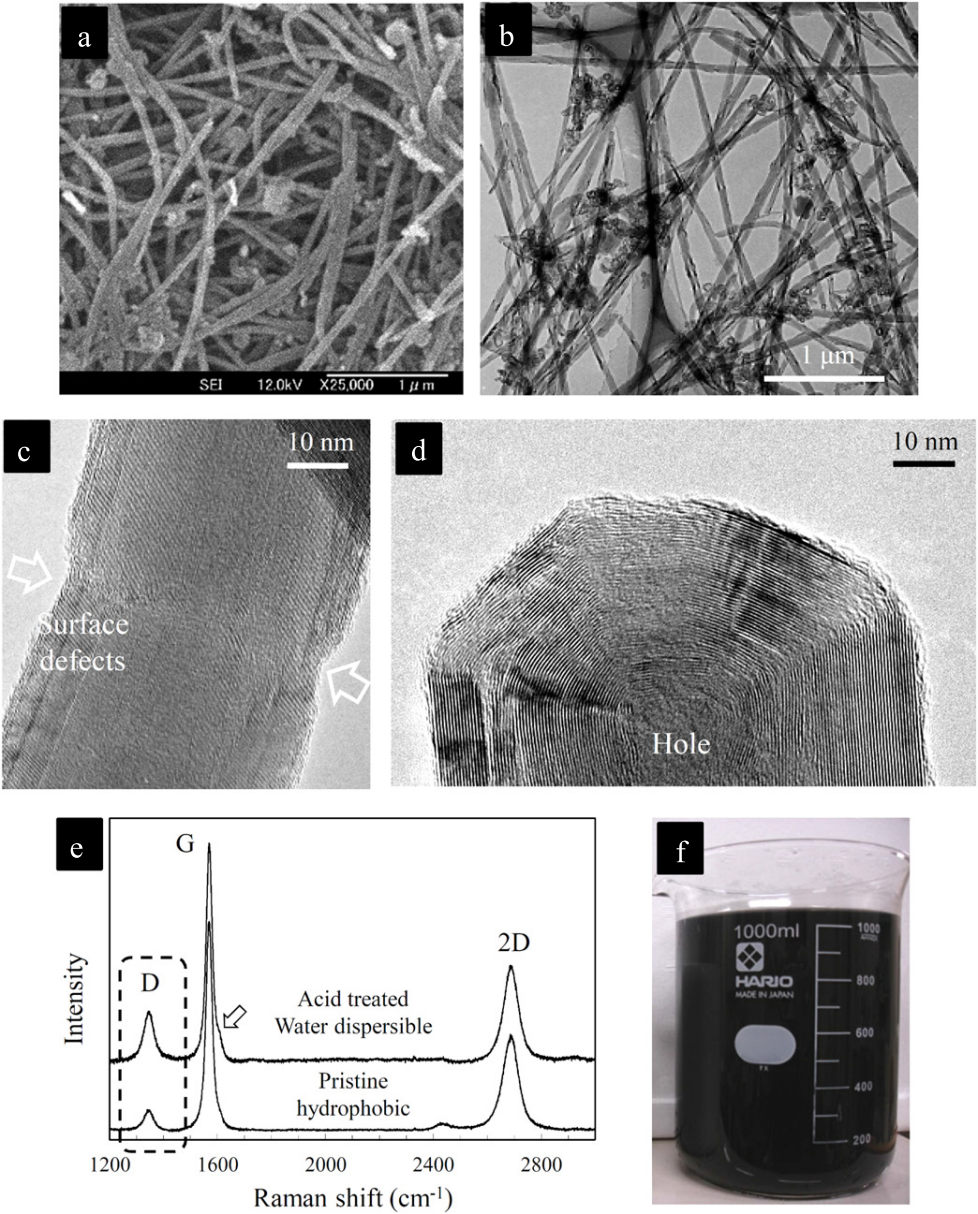
(a) SEM image; (b) TEM image; (c) high-resolution TEM (HRTEM) image of the length; (d) HRTEM image of the tip; and (e) Raman spectrum of the water dispersible, slightly surface-defective, crystalline MWCNTs prepared by a controlled acid-treatment process [17]. The Raman shoulder at ∼1620 cm^−1^ is attributed to the carboxylic groups attached to the surface of the MWCNTs responsible for their superb colloidal stability in water [17]. (f) The typical appearance of the aqueous suspension of these hydrophilic MWCNTs containing well-dispersed individual MWCNTs (pH ∼ 3–4), which are stable over months. Such stable aqueous suspensions were used as the precursor to fabricate the intended ‘clean’ MWCNT–ceramic composite system [17, 44]. Reprinted from M Estili *et al* 2008 *Acta Mater*. **56** 4070 (Copyright 2008 with permission from Elsevier) and M Estili *et al* 2012 *Adv. Mater*. **24** 4322 (Copyright © 2012 WILEY-VCH Verlag GmbH & Co. KGaA, Weinheim).

**Figure 3.**
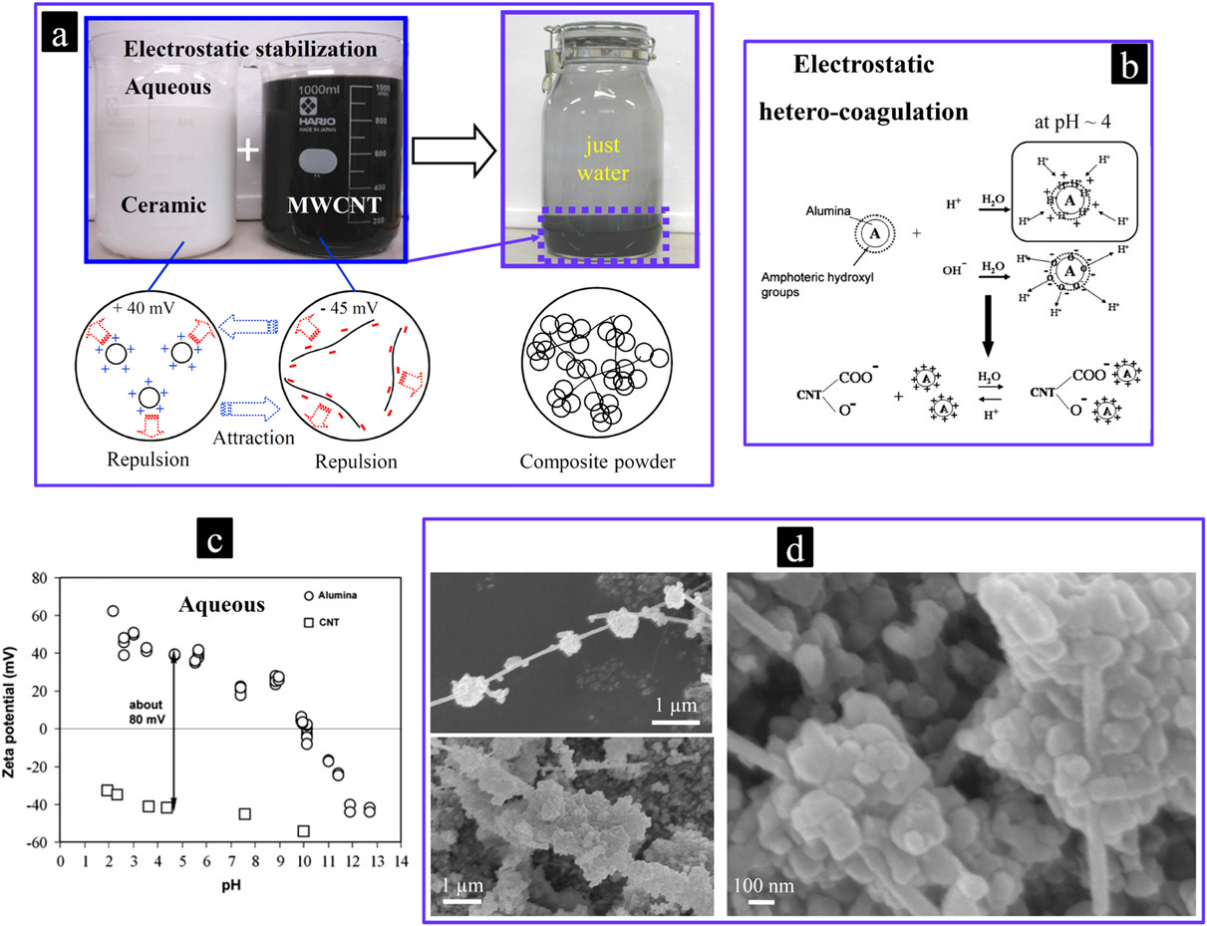
(a) Scalable, aqueous electrostatic hetero-coagulation method employed to uniformly disperse the individual MWCNTs within the ceramic powder without using any surfactants or polymers; (b) the coagulation mechanism involved; (c) Zeta potential-pH responses of the acid-treated MWCNTs and *α*-Al_2_O_3_ powder in water; and (d) SEM images of some individual MWCNTs decorated by the ceramic particles. The ceramic particles arresting and immobilizing the individual MWCNTs due to electrostatic attractive forces would prevent the common re-agglomeration of the MWCNTs during the water-removal step and during drying, which ensures the uniform MWCNTs dispersion within the bulk ceramic matrix after the sintering process [17, 44, 46]. Reprinted from M Estili *et al* 2008 *Acta Mater*. **56** 4070 (Copyright 2008 with permission from Elsevier), M Estili *et al* 2012 *Adv. Mater*. **24** 4322 (Copyright © 2012 WILEY-VCH Verlag GmbH & Co. KGaA, Weinheim) and M Estili *et al* 2008 *Scr. Mater*. **58** 906 (Copyright 2008 with permission from Elsevier).

**Figure 4.**
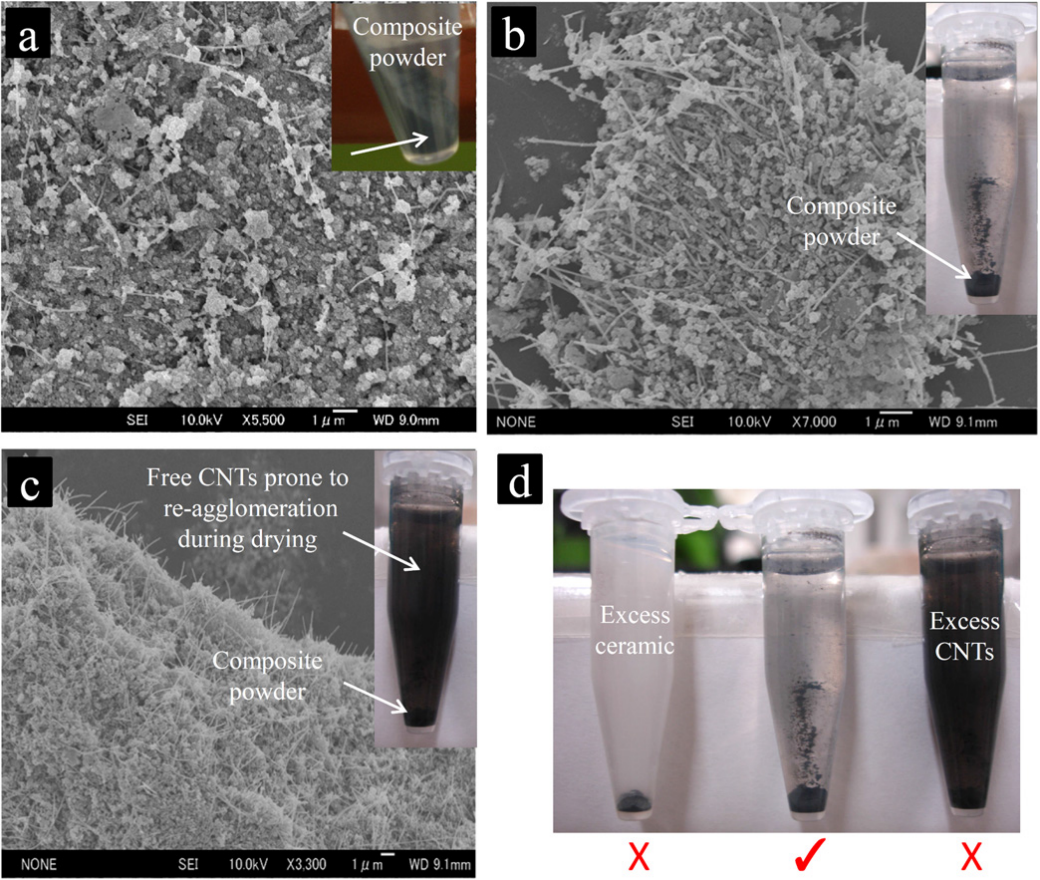
SEM images of the composite powders prepared with low-to-high MWCNT concentrations. (a) 3.5 vol %; (b) 16 vol %; and (c) 22 vol % CNT concentrations. The insets demonstrate the centrifuged cells, which could be used to qualitatively evaluate the strength and irreversibility of the CNT–ceramic particles’ attraction during the hetero-coagulation process and to confirm the existence of the possible non-arrested, free MWCNTs in the composite powder prone to re-agglomeration during the water-removal and drying processes. The dried composite powders were sonicated in water for an hour prior to the centrifuge process (1 min at 6200 rpm). (d) The three possible centrifuge-cell results with different supernatants; only the composite powders, for which the supernatants become transparent, were recommended for the subsequent drying and sintering [46]. Reprinted from M Estili *et al* 2008 *Scr. Mater*. **58** 906 (Copyright 2008 with permission from Elsevier).

**Figure 5.**
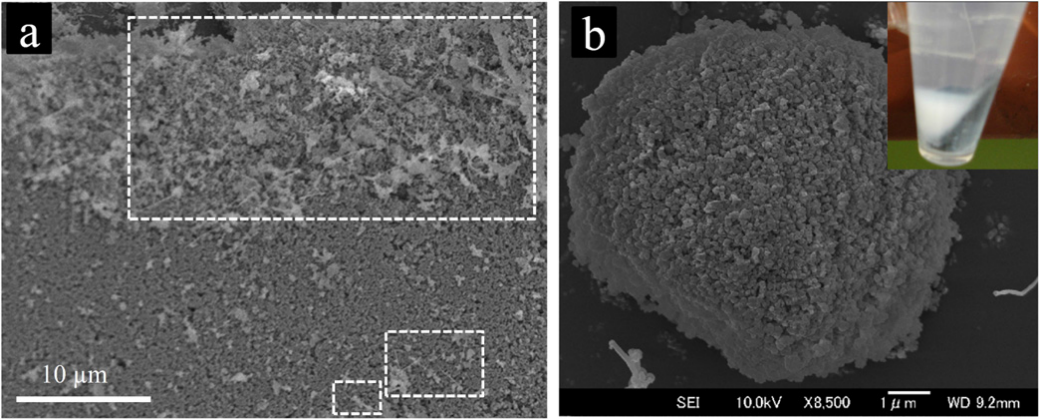
SEM images of the composite powder containing pristine hydrophobic MWCNTs and alumina powders, which were prepared by the conventional ball milling process in ethanol in our group. (a), (b) Non-uniform distribution of the pristine MWCNTs within the ceramic powders with agglomerations seen in the highlighted areas [17]. No CNTs could be observed within the ceramic powder in (b). The inset in (b) shows the corresponding centrifuge cell, confirming the poor connection between the hydrophobic CNTs and the ceramic particles. Reprinted from M Estili *et al* 2008 *Acta Mater.* **56** 4070 (Copyright 2008 with permission from Elsevier).

The processing obstacles were resolved by establishing a scalable, aqueous colloidal (electrostatic hetero-coagulation) approach able to decorate gram-level amounts of surface-functionalized, negatively charged, individual MWCNTs uniformly dispersed in water, with positively charged *α*-Al_2_O_3_ nanoparticles also dispersed in water, using purely electrostatic forces (figures 1–3) [17, 44, 46]. Hydrophilic, carefully acid-surface-treated MWCNTs with high crystallinity but slight surface defects [17] (for an enhanced compatibility and CNT–ceramic interfacial shear resistance) and commercially available, high-quality TM-DAR grade *α*-Al_2_O_3_ powders have been used in these studies. This novel process totally prevents the facile re-agglomeration of MWCNTs during the water-removal step and during drying, as they are all arrested and immobilized by the ceramic nanoparticles prior to the water removal/drying (thus, no free, undecorated CNTs then exist for the commonly observed, secondary re-agglomeration), which ultimately guarantees the uniform dispersion of the CNTs within the bulk ceramic matrix after the sintering process. This method could be used to synthesize composite powders with a wide range of CNT concentrations (∼2–20 vol %) and is able to determine whether or not some non-arrested and undecorated CNTs leading ultimately to the formation of porosity in the sintered composite bulk remain in the composite powder simply by looking at the transparency of the supernatant of the final stagnant mixture (figure 3) or the sonicated-centrifuged cells (figure 4) [46]. In order to understand the success of this method and emphasize the importance of the usage of hydrophilic MWCNTs (figure 2) (instead of pristine hydrophobic ones (figure 1)), the composite powder prepared by the conventional wet ball milling process is shown in figure 5. The distribution of the MWCNTs within the ceramic powders is very non-uniform, with many agglomerations. The centrifuge cell shown in the inset also confirms the poor connection between the hydrophobic CNTs and the hydrophilic ceramic particles. The sintering of these composite powders would lead to a porous bulk with degraded mechanical properties.

**Figure 6.**
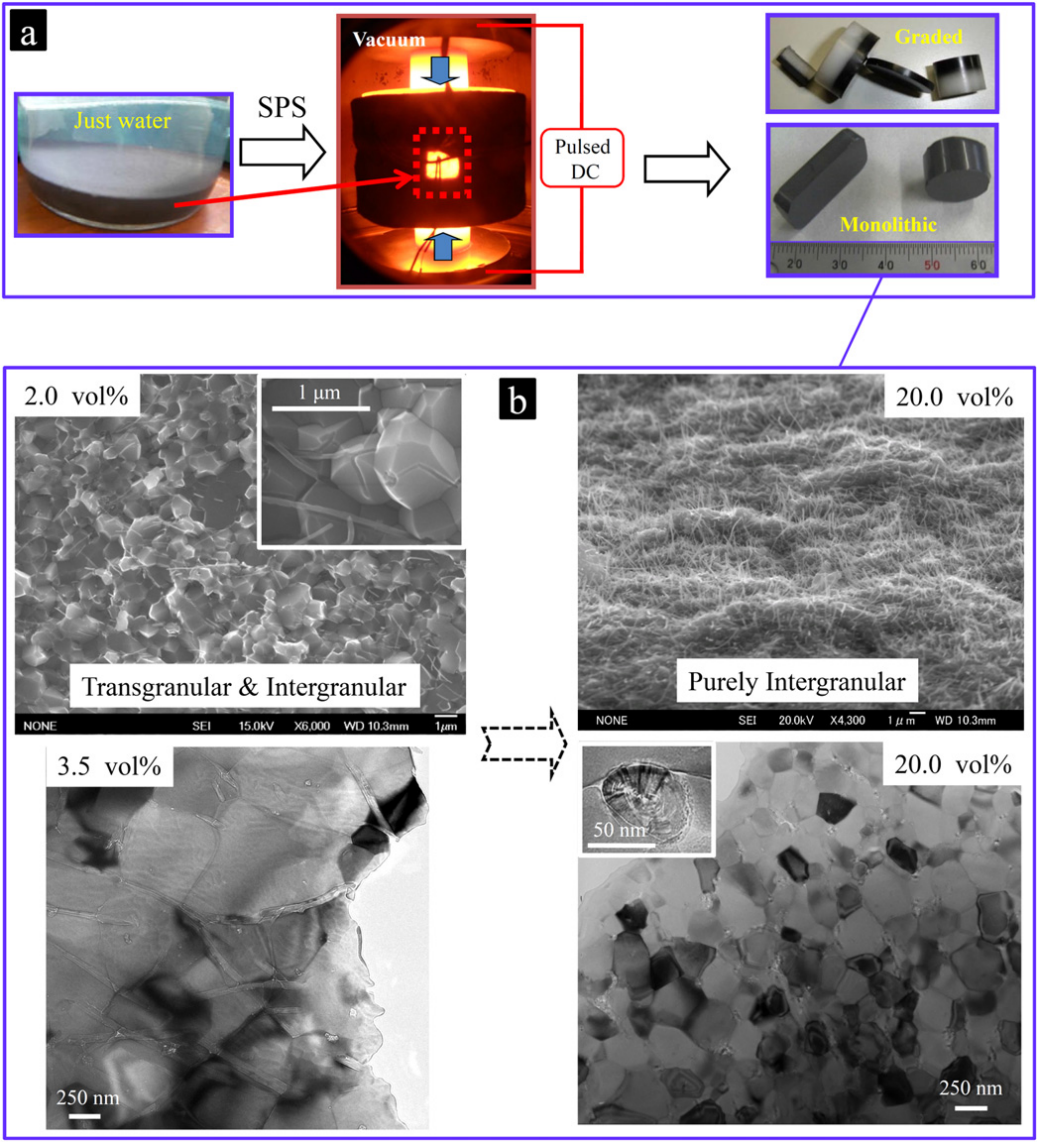
(a) Consolidation of the prepared composite powders into monolithic or novel graded FGM composites using the fast SPS sintering technique in vacuum and at a temperature of ∼1300 °C; and (b) SEM (fracture surface) and TEM images (polished surface) of the composites with low and high MWCNT concentrations, which demonstrate the important effect of the MWCNT content on the microstructure and fracture mode of the ceramic matrix. The inset in the 20 vol % TEM image shows an individual MWCNT (from its radial direction) in intimate contact with the matrix, which seems to be compressed and buckled [1, 17, 44, 49]. Reprinted from M Estili *et al* 2013 *Nanotechnology* **24** 155702, M Estili 2008 *Acta Mater*. **56** 4070 (Copyright 2008 with permission from Elsevier), M Estili 2012 *Adv. Mater*. **24** 4322 (Copyright © 2012 WILEY-VCH Verlag GmbH & Co. KGaA, Weinheim) and M Estili *et al* 2010 *Mater. Sci. Forum* **631–632** 225.

**Figure 7.**
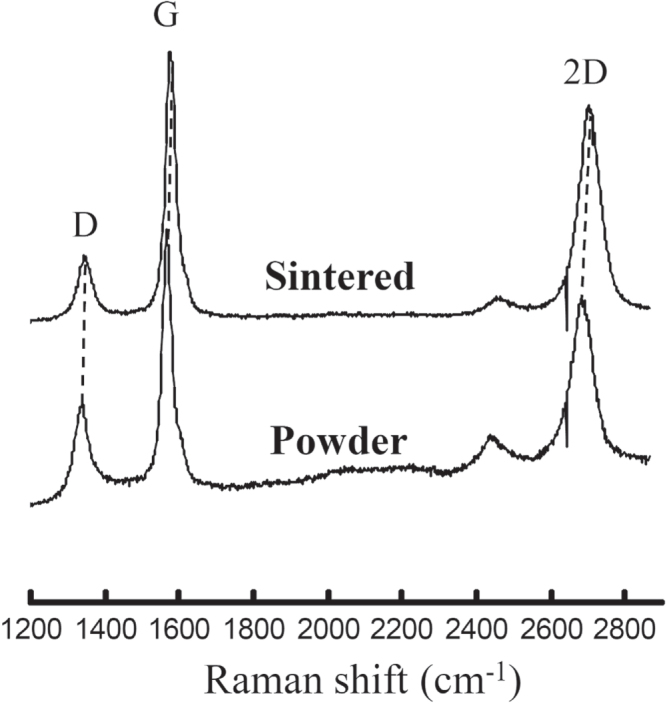
Raman spectra of the MWCNTs before and after the sintering process, which confirms no damage to their structures. Note the comparable I_D_/I_G_ ratio in both MWCNTs [44]. Reprinted from M Estili *et al* 2012 *Adv. Mater*. **24** 4322 (Copyright © 2012 WILEY-VCH Verlag GmbH & Co. KGaA, Weinheim).

Next, by employing a rapid pressure and pulsed dc-current-assisted densification process (commonly known as pulsed electric-current-assisted sintering or spark plasma sintering (SPS)) [53–60], fully dense monolithic [1, 17, 44] or graded CNT–Al_2_O_3_ ceramic composite bulks with low-to-high MWCNT concentrations [48, 49] have been fabricated (figure 6). Regardless of the concentration, the high-quality MWCNTs are individually and uniformly distributed on the grain boundaries of the structurally uniform, pore-free Al_2_O_3_ matrix, with intimate physical interfaces and random orientations (figures 6 and 7). The important effect of the MWCNT content on the microstructure and fracture mode of the ceramic matrix could also be clearly observed. These novel composites could be used as standard samples to properly investigate the reinforcing ability (and possible mechanisms) of the CNTs in a ceramic matrix. On the other hand, with the challenging issues of (1) dispersion, (2) interface, (3) CNTs post-sintering quality and (4) mass-production, which are fully addressed using such a versatile and scalable method [44], the true effect of the MWCNT concentration on the macroscopic mechanical performance of the composite—especially in such a pore-free and structurally uniform matrix platform—could also be studied for the first time; the results will be reviewed in section 5.

The possibility of using high-quality precursors and the precise control of the CNT concentration of the composite and the mass-production of homogeneous composite powders (even in high CNT concentrations) are merits of this scalable approach, which are impossible to achieve by the conventional and *in situ* growth-based methods. Recently, this method was also shown to be successful in uniformly dispersing the 2D graphene oxide nanosheets in a tetragonal ZrO_2_ ceramic matrix for thermoelectric applications [55]. It is clear that by using this cost-effective and scalable approach, CNT, graphene, carbon fiber or any other 1D/2D nanostructure-containing composites with different matrix materials designed for a variety of applications could also be successfully fabricated, once an attractive electrostatic or electrosteric force simply prevails between their modified surfaces.

## 3. Functionally graded CNT–ceramic composites

**Figure 8.**
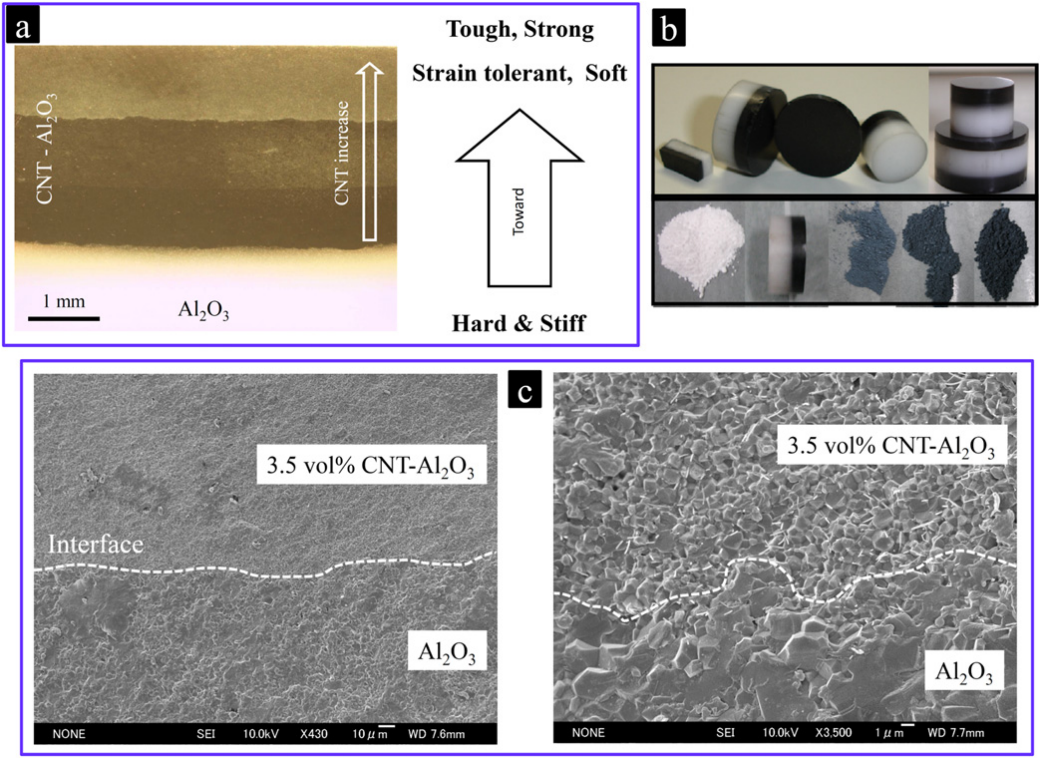
The use of MWCNTs to fabricate functionally graded Al_2_O_3_ ceramic. (a) Using the concept of FGM, CNT-concentrated ceramics, which are softer, tougher, stronger and more strain tolerant, could be joined—without any premature failure—to the pure ceramic or low-CNT-concentration ceramic matrix composites, which are harder and stiffer; (b) photos of the SPSed FGMs; and (c) SEM images showing the flawless interface between the monolithic Al_2_O_3_ and 3.5 vol % CNT–Al_2_O_3_ composite at different magnifications [48, 49]. Reprinted from M Estili *et al* 2008 *Scr. Mater*. **59** 703 (Copyright 2008 with permission from Elsevier) and M Estili *et al* 2010 *Mater. Sci. Forum* **631–632** 225.

In another pioneering work, synthesized homogeneous composite powders with different MWCNT concentrations were used to fabricate MWCNT-based functionally graded ceramics with in-depth graded properties and functionalities that are promising for a variety of challenging structural, electronic and bio-related applications [48, 49]. This has been the first time that CNTs have been used as a grading agent to fabricate functionally graded materials (FGMs). The concept of FGMs, proposed in Japan for the first time, has been inspiring researchers worldwide to effectively manipulate and combine irreconcilable properties and functionalities of conventional materials to fabricate a new generation of advanced composites for novel applications [61–70]. Bearing less compromise between the constituents’ properties by providing in-depth graded compositions, microstructures and properties, the FGMs have offered good promise in structural, electronic and biomaterial applications [61–76]. The concept of FGMs could be successfully employed to fabricate graded ceramic-matrix composite bulks containing low-to-high concentrations of CNTs with tailored graded properties, which are promising for a variety of novel applications, especially those that structurally demand a hard shell and a tough core. Accordingly, CNT-concentrated ceramics, which are softer, tougher, stronger and more strain tolerant [1, 44], could be joined—without any premature failure [70]—to the pure ceramic or low-CNT-concentration ceramic matrix composites, which are harder and stiffer (figure 8) [1, 17, 48]. The success in processing the CNT–ceramic FGMs inspired research on CNT-metal [71–73] and CNT/carbon fiber-polymer FGMs [74–76] for various applications. The CNTs have indeed proved to be a novel grading agent to realize multifunctional graded materials.

## 4. Direct characterization of the load carrying capacity of CNTs embedded in a ceramic matrix

The mechanical response of a pore-free and structurally uniform CNT–ceramic composite strongly depends on the load carrying capacity of CNTs, which is determined first by the amount of load transfer at the interface and second by the intrinsic load bearing ability of CNTs after incorporation into the ceramic matrix. If the load transfer is poor, or the load bearing ability of CNTs is possibly reduced during the processing, which generally involves high-temperature and surface treatments, the CNTs would act as a defect and thus lower the mechanical properties even if they are uniformly dispersed within the matrix with intimate interfaces [9, 10]. Therefore, direct characterizations of the load transfer, as well as the load bearing ability of CNTs while embedded in the ceramic matrix, are of great importance in understanding the reinforcing ability and mechanism of CNTs in a ceramic material. In this section, we briefly review the recent efforts in directly characterizing the load carrying capacity and energy dissipation of MWCNTs intimately embedded in a host Al_2_O_3_ matrix, which address the first- and second-raised fundamental questions mentioned in the Introduction.

**Figure 9.**
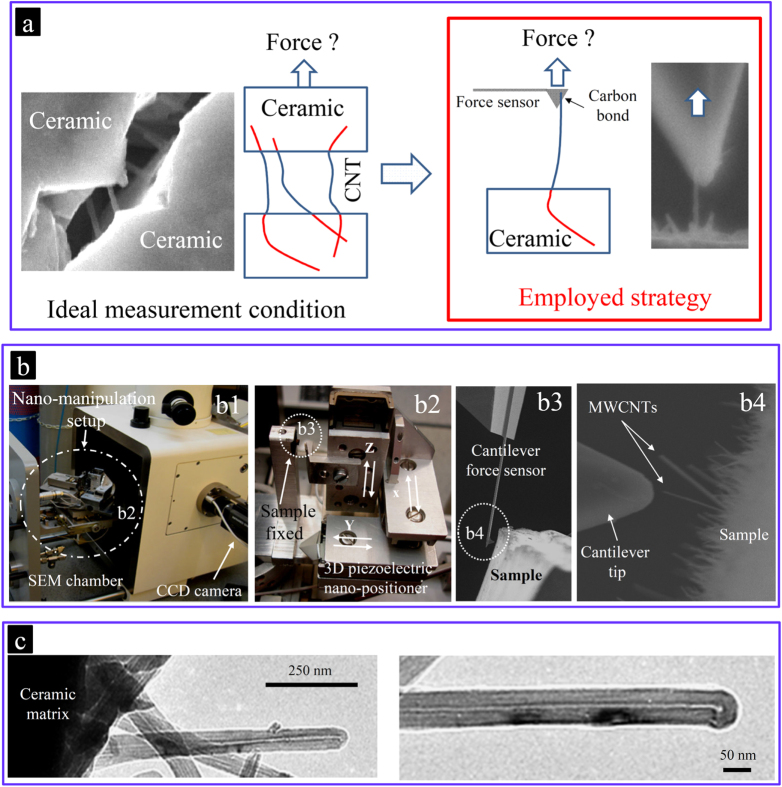
Estimation of the load carrying capacity of the MWCNTs when embedded in the ceramic matrix. (a) the *in situ* pullout strategy; (b) nano-manipulation equipment; and (c) typical TEM images of some totally crack-deflecting, undamaged MWCNTs exposed on the fracture surface of the composite, which are suitable for such *in situ* pullout and tensile measurements (similar to those shown in figure 10(b)). The embedded parts of the CNTs are shown in red to emphasize the existence of the radial compressive stresses applied from the ceramic matrix. The load carrying capacity is determined by the combined contributions of the interfacial shear resistance as well as the intrinsic load bearing ability of the MWCNTs, when embedded in the ceramic matrix. The weakness of any of these two parameters would weaken the overall load carrying capacity even if the other parameter is superior [9, 10, 17]. Reprinted from M Estili and A Kawasaki 2010 *Adv. Mater.* **22** 607 (Copyright © 2010 WILEY-VCH Verlag GmbH & Co. KGaA, Weinheim), M Estili *et al* 2011 *J. Mater. Chem.* **21** 4272 (reproduced by permission of The Royal Society of Chemistry (RSC)) and M Estili 2008 *Acta Mater*. **56** 4070 (Copyright 2008 with permission from Elsevier).

**Figure 10.**
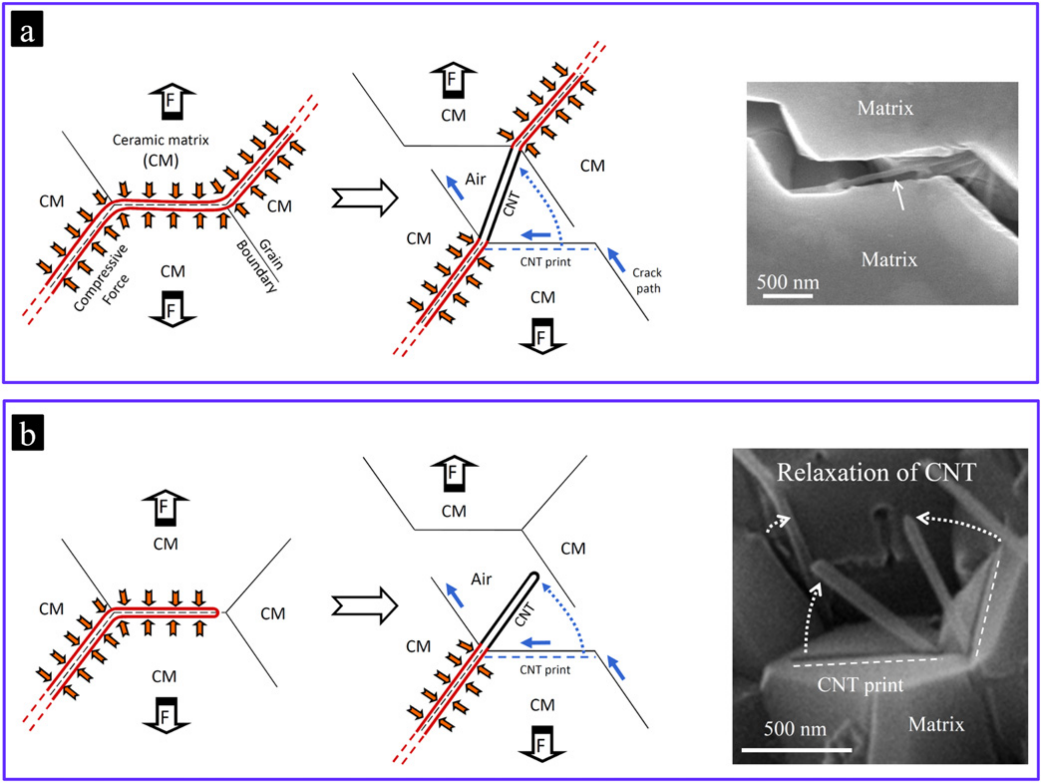
Schematics and real examples (SEM images) of the two possible interactions between a propagating crack in the ceramic matrix and the individual MWCNTs in a randomly oriented CNT–ceramic matrix composite system. The red lines and arrows correspond to the radial compressive stress applied from the ceramic matrix [9]. (a) Demonstration of the interaction in which the CNTs crossing the upper and lower crack surfaces are partially detached from the matrix and tensile-loaded mostly in a deflected configuration upon further crack opening. These CNTs are able to dissipate energy during loading only if the shear resistance at the CNT–ceramic interface is strong enough to avoid their easy pullout from the matrix; and (b) the demonstration of the interaction in which the non-crossing CNTs lying partially on the crack surface are detached from the matrix and then return to their original relaxed figures upon further crack opening without being loaded. These totally crack-deflecting CNTs are unable to dissipate energy during loading but, they degrade the mechanical properties of the composite [10, 17]. Reprinted from M Estili *et al* 2011 *J. Mater. Chem*. **21** 4272 (reproduced by permission of The Royal Society of Chemistry (RSC)) and M Estili 2008 *Acta Mater*. **56** 4070 (Copyright 2008 with permission from Elsevier).

**Figure 11.**
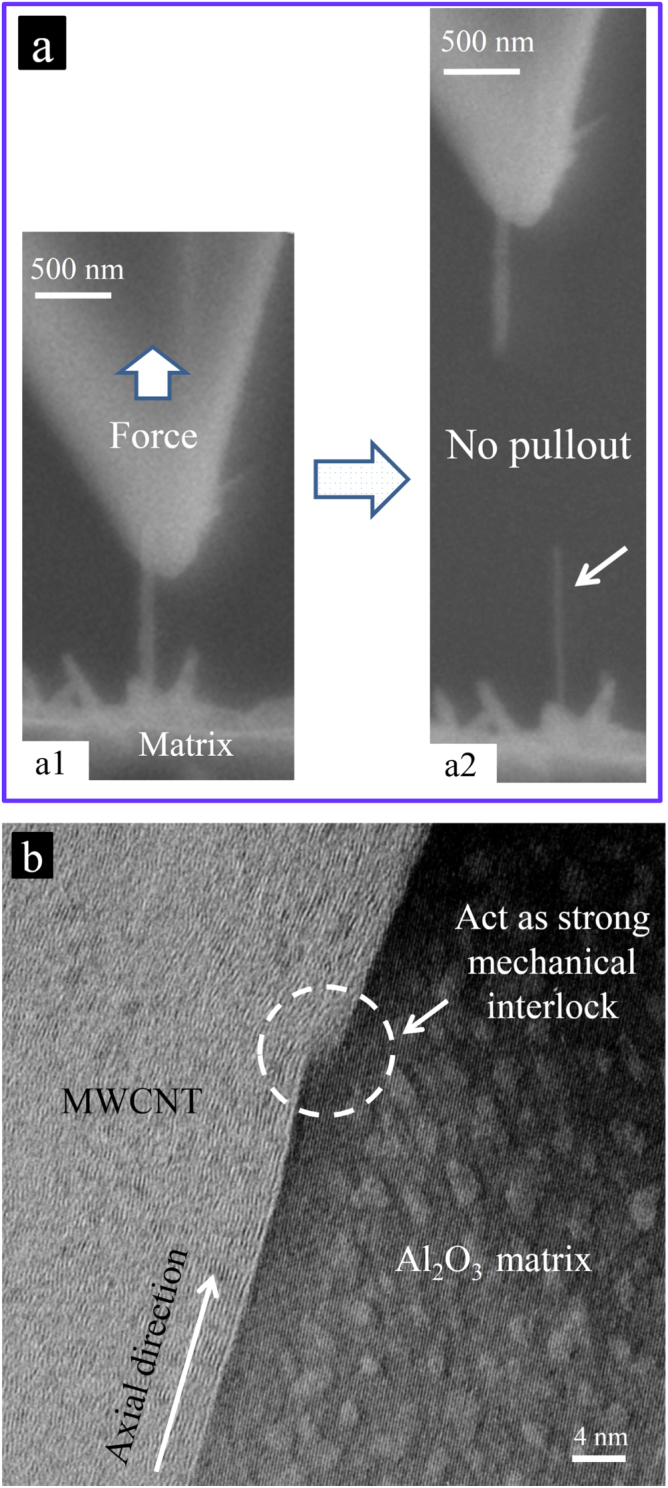
The typical pullout experiment. (a) SEM images of an individual MWCNT under tensile loading, which, at the moment right after its failure, confirms no CNT pullout from the ceramic matrix. The rather large differences in the diameters of the two broken parts suggest the failure of many walls simultaneously, which indeed could dissipate more energy than the common one-wall failures reported in the literature. (b) HRTEM image of the CNT–Al_2_O_3_ interface, where the nanoscale surface-defect of the MWCNT is completely filled with the ceramic matrix, causing strong interfacial shear resistance during loading, which prevents the MWCNT from being pulled out from the matrix; this is regarded as the ultimate load transfer to the MWCNT from the ceramic matrix [9]. Reprinted from M Estili M and A Kawasaki 2010 *Adv. Mater*. **22** 607 (Copyright © 2010 WILEY-VCH Verlag GmbH & Co. KGaA, Weinheim).

**Figure 12.**
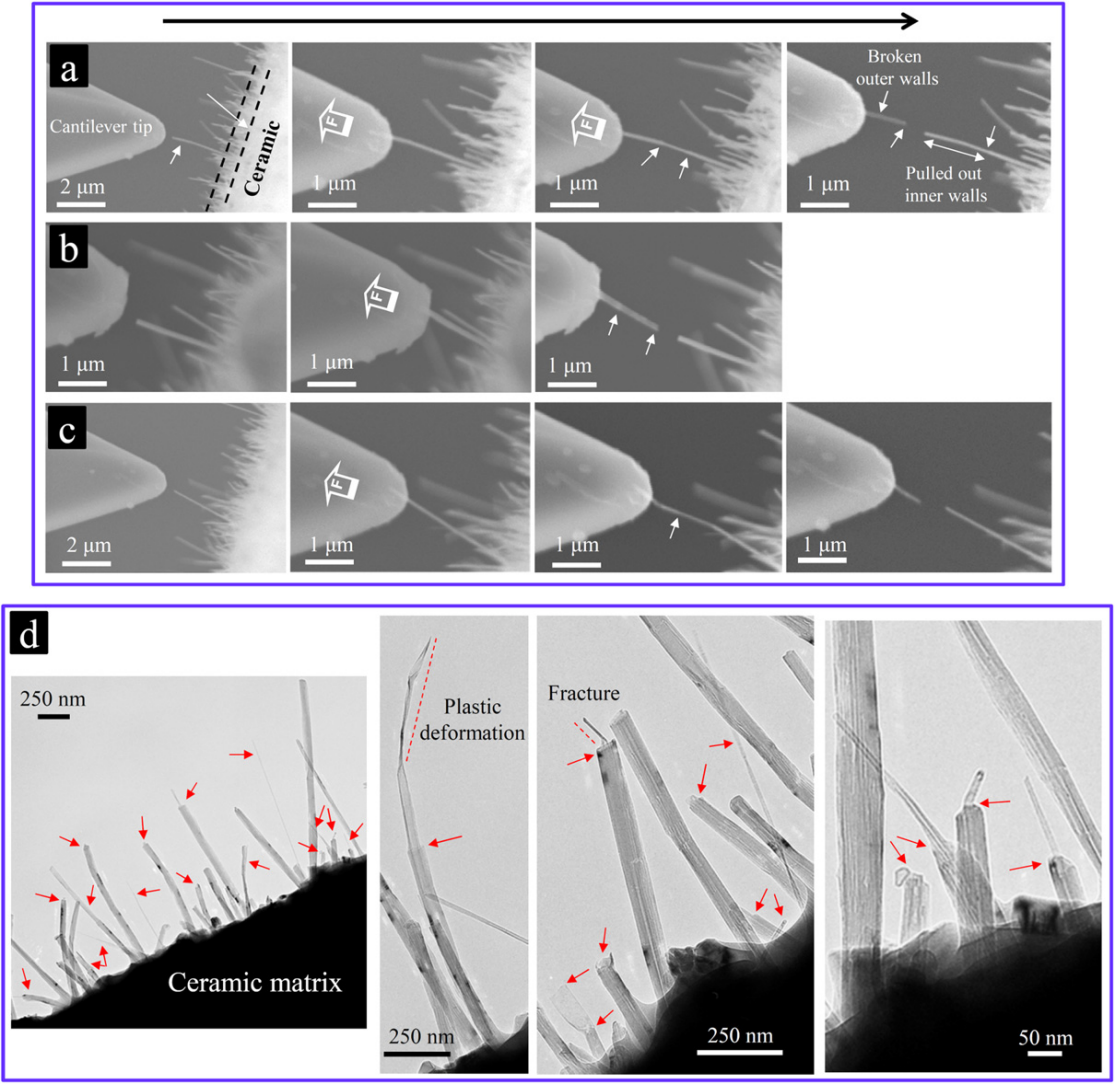
Highly efficient energy dissipation by the MWCNTs’ failures during loading. (a), (b), (c) Various highly energy-dissipating failures and plastic buckling of the MWCNTs occurred during the *in situ* pullout experiments (SEM images); and (d) during the fracture of the composite (typical TEM images of the fracture surface) [1, 10]. Reprinted from M Estili *et al* 2013 *Nanotechnology* **24** 155702 and M Estili *et al* 2011 *J. Mater. Chem.* **21** 4272 (reproduced by permission of The Royal Society of Chemistry (RSC)).

**Figure 13.**
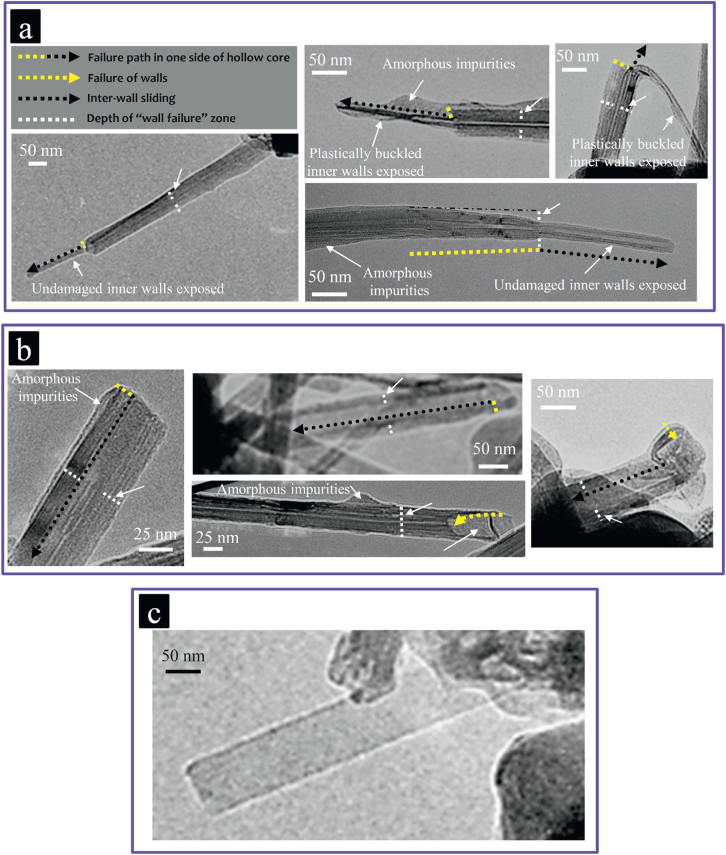
TEM images of the fragments of several fractured MWCNTs (multiwall-type failures), which could be observed on the fracture surface of the ‘clean’ composite [9]. (a) ‘Sword’ part; (b) multiwall ‘sheath’ part; and (c) few-wall ‘sheath’ part. Reprinted from M Estili and A Kawasaki 2010 *Adv. Mater*. **22** 607 (Copyright © 2010 WILEY-VCH Verlag GmbH & Co. KGaA, Weinheim).

The load transfer phenomenon at the intimate CNT/ceramic interface is governed by an interfacial shear mechanism that originated from the mechanical interlocking of structural inhomogeneity and defects of the CNTs with the matrix [9] and/or the formation of chemical bonding at the interface. The higher the interfacial shear resistance, the higher the extent of the load transfer from the matrix to the CNTs, which could even exceed the failure resistance of the CNTs. Addressing questions (1) and (2), the strategy to evaluate the interfacial load transfer was to perform direct pullout experiments for the CNTs exposed on the fracture surface of the composite, as shown in figure 9. Note that the observation of CNTs on the fracture surface or while crossing the microcracks does not necessarily imply that their pullout process has been frictional and energy-dissipating and thus does not necessarily prove the load transfer to the CNTs. These features could have also occurred without friction and/or relaxation of the CNTs, which lied partially or entirely on the crack planes without crossing. On the fracture surface, there exist many CNTs lying on the crack planes, which only deflected the crack without being loaded. Such unloaded CNTs could be selected for the pullout experiments (figure 10(b)). Estili and Kawasaki used a simple measurement and nano-manipulation system, which was installed in the chamber of a scanning electron microscope (SEM) [9, 10]. This system included a nanomanipulator equipped with a cantilever as a highly sensitive force sensor. It was used to directly characterize the interfacial shear resistance as well as the mechanical response of individual MWCNTs embedded intimately in the *α*-Al_2_O_3_ ceramic matrix. A schematic of the measurement system is shown in figure 9. The pullout experiments have been performed on about 150 exposed MWCNTs in different parts of the composite’s fracture surfaces. Absolutely no CNT pullout, either frictional or non-frictional, was observed; instead, they realized unprecedented failures of the MWCNTs, which could generally be highly energy-dissipating (figures 11, 12 and 13). These strongly suggest the existence of strong interfacial shear resistance, which exceeded even the failure resistance of the MWCNTs, i.e. complete transfer of the applied load from the ceramic matrix to the MWCNTs. The absence of pullout and the observation of failure (ultimate load transfer to the CNTs until they eventually fail) indeed reflect a new energy dissipating process at the nanoscale, which was not reported previously for any CNT-reinforced composites.

**Figure 14.**
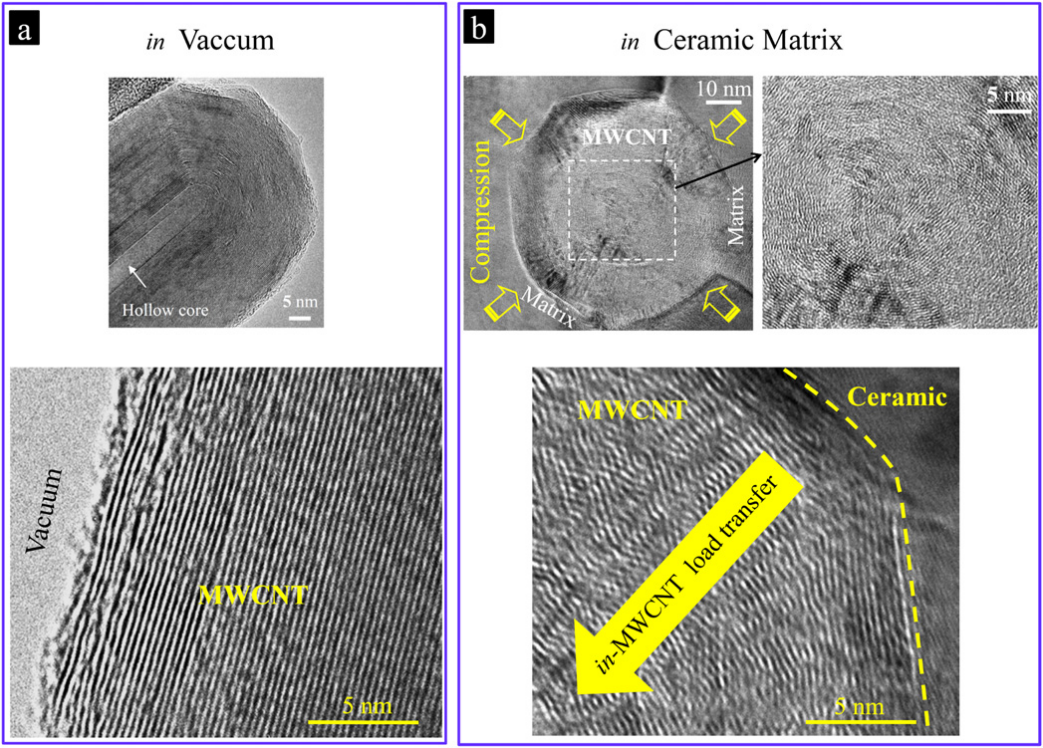
The mechanism behind the highly energy-dissipating multiwall-type failures of the MWCNTs during loading. (a) HRTEM images of an individual MWCNT in a vacuum environment, highlighting the tube’s hollow core, as well as the parallel, straight inner walls, with no obvious interconnections between the walls; and (b) HRTEM images showing the structural evolution (elastic buckling) of an individual MWCNT when embedded in the compressive-stressing ceramic matrix, which results in the formation of in-wall irregularities transferring the load from the outermost walls to the generally unloaded inner walls and thus enhancing the MWCNT’s maximum sustainable load [9]. Reprinted from M Estili and A Kawasaki 2010 *Adv. Mater*. **22** 607 (Copyright © 2010 WILEY-VCH Verlag GmbH & Co. KGaA, Weinheim).

After the qualitative pullout experiments, a quantitative estimation of the extent of energy dissipation during the observed unprecedented MWCNTs failures (figures 12 and 13), which directly depends on the load bearing ability of the MWCNTs after incorporation into the ceramic matrix, was planned. As mentioned earlier, the load bearing ability of CNTs when embedded in a ceramic matrix could be different from those outstanding ones that were reported, which typically belong to the MWCNTs loaded in a transmission electron microscope (TEM) vacuum environment [5, 6]. Embedding the CNTs, for instance, into the alumina ceramic using the SPS process requires a high temperature generally exceeding 1200 °C and pressure, which could possibly lead to the formation of defects and to the structural evolution in the CNTs. On the other hand, after sintering and the formation of intimate interfaces, a uniform radial compressive stress could be applied to the CNTs from the ceramic matrix due to their differences in Young’s moduli and their coefficients of thermal expansion, which could further enhance their structural changes [9, 10, 26] (figure 14).

**Figure 15.**
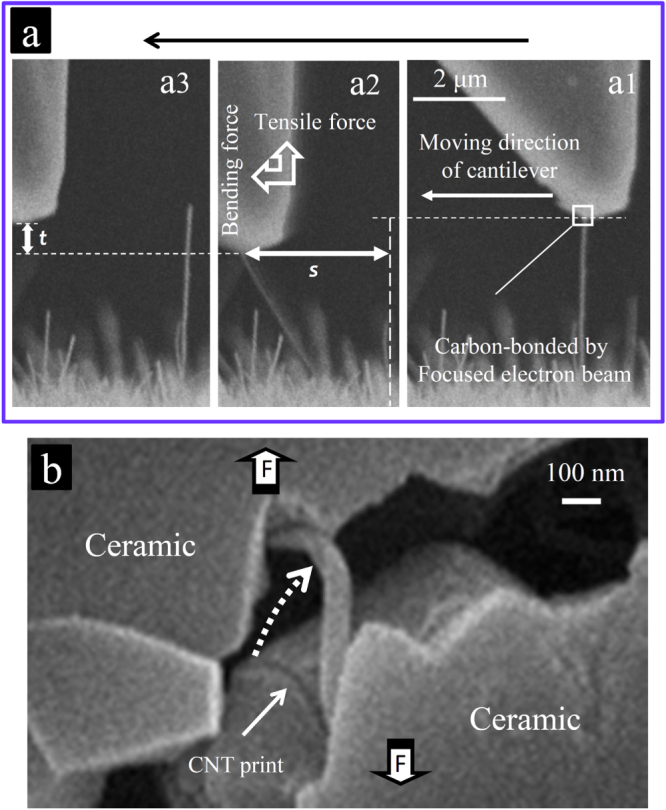
Estimation of the load bearing ability of the MWCNTs under a combined tensile-bending loading configuration. (a) SEM images describing the *in situ* loading technique able to apply considerable amount of tensile and bending loads simultaneously: (a1) the MWCNT is carbon-bonded to the cantilever tip from the side prior to the loading; (a2) MWCNT under simultaneous tensile and bending loads right before failure of the carbon bond; and (a3) despite experiencing the large combined tensile-bending loads, the MWCNT does not fail and returns to its original relaxed figure once the carbon bond fails (*s* and *t* are deflections of the MWCNT and cantilever, respectively). (b) The real example showing a crack-bridging MWCNT under combined tensile and bending loads (similar to the one shown in figure 10(a)) [10]. Reprinted from M Estili *et al* 2011 *J. Mater. Chem*. **21** 4272 (reproduced by permission of The Royal Society of Chemistry (RSC)).

In order to quantitatively (but roughly) estimate the energy dissipation during the MWCNTs’ failures while crack bridging, the maximum sustainable loads of several MWCNTs exposed on the fracture surface were measured using the cantilever force sensor (figure 9) and compared with those of near-perfect arc-discharge-grown MWCNTs experimentally tested in a vacuum environment of a TEM. The measurements were performed in tensile (figures 11 and 12) as well as combined tensile-bending loading modes (figure 15). In general, the CNTs embedded in a ceramic matrix experience a combination of tensile and bending loads during the bridging of the cracks of the ceramic matrix (figure 10). According to a study by Wong *et al* [8], MWCNTs (in air or vacuum) possess dramatically less strength and stiffness in the bending mode, which might seriously limit their applications as a reinforcing agent. Therefore, such a possibility was investigated while the MWCNTs were partially embedded in the ceramic matrix (figure 15) as well. Note that the precise quantitative measurement of the load bearing ability of the CNTs in the ceramic matrix (under the effect of the compressive-stressing ceramic matrix) requires that both ends of the MWCNTs are embedded in the matrix, as shown in figure 9. Perhaps, the preparation of such a loading configuration is impractical; thus, an alternative way was used, though it provided only the lower limit of the true load bearing ability [9, 10].

In the tensile loading mode, the cantilever tip was simply carbon-bonded [77] from the side to the MWCNTs’ tip and then retracted until the MWCNTs’ failure. The cantilever’s deflection right before the failure was multiplied by the cantilever’s spring constant to calculate the maximum sustainable load (figures 9, 11 and 12) [9, 10]. For the combined tensile-bending loading mode, a more complex strategy was employed, as shown in figure 15 [10]. In brief, the cantilever tip was carbon-bonded to the selected MWCNT and then moved preferentially perpendicular to the MWCNT’s axis until the failure of the carbon bonding; the maximum bending and tensile loads applied to the MWCNTs right before the failure of the carbon bonding were finally calculated. In this loading mode, the carbon-bond strength and the angle between the CNT axis and cantilever’s moving direction could determine the fraction of tensile load and bending deflection, which are simultaneously applied to an individual CNT. In order to apply a large deflection combined with a considerable tensile load prior to the carbon-bond break, a suitable angle (∼90°) and a strong carbon bond are required; otherwise, the carbon bond might fail easily, or undesired loading configurations, such as a large deflection/small tensile load or a small deflection/large tensile load, might occur. However, it has been challenging to find suitable CNTs on the fracture surface with suitable orientations for the realization of the desired loading configuration, considering the random orientation of the CNTs and the 2D field of view of the SEM. Therefore, the suitability of many CNTs for this set-up had to be examined by a trial and error process; ultimately, only two MWCNTs were found for which the desired loading configuration was established. Then, using a simple hollow beam theory [78, 79], assuming the MWCNTs as an elastic isotropic beam, the maximum bending strains and stresses applied to the MWCNTs were calculated after measuring the deflection (*s* in figure 15) and geometry of the MWCNTs from the SEM images [10]. Next, the maximum tensile load applied to the MWCNTs right before the failure of the carbon bonding was calculated by multiplying the total vertical cantilever deflection (*t* in figure 15) to the spring constant of the cantilever and to a function that includes the deflection angle of the MWCNT and offset angle of the cantilever.

**Figure 16.**
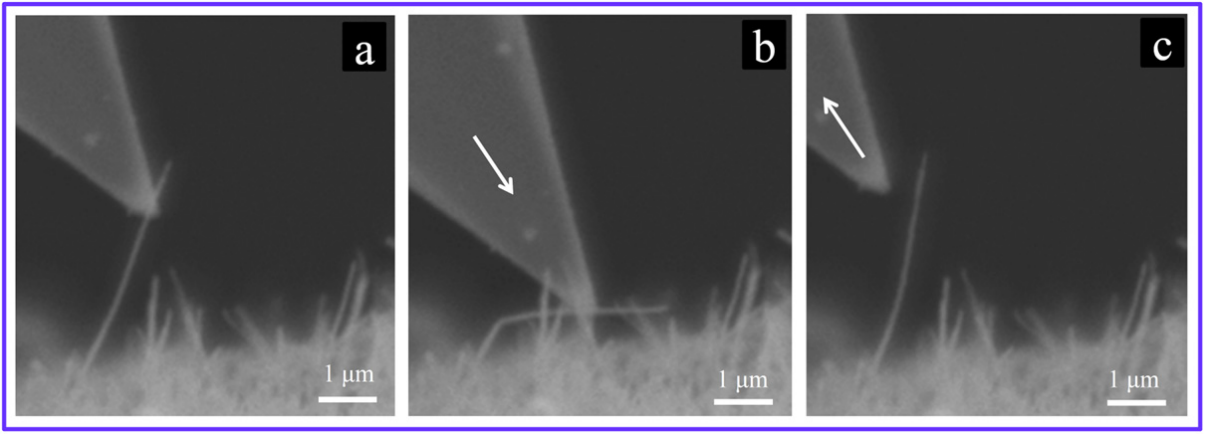
Typical *in situ* bending tests examining the flexibility of the MWCNTs after incorporation into the ceramic matrix. (a) Before bending; (b) during bending; and (c) after unloading and relaxation of the MWCNT. The white arrows show the moving direction of the cantilever tip. The MWCNTs remain highly flexible, even after incorporation into the ceramic matrix and are able to accommodate a large amount of elastic strain without plastic deformation or failure.

Surprisingly, in both loading modes, despite the harsh processing condition experienced by MWCNTs, their lower bound load bearing ability in the ceramic matrix was estimated to be remarkably superior to even those of near-perfect, straight, arc discharge-grown MWCNTs tested in vacuum [5, 6]. Furthermore, the exceptional flexibility of MWCNTs after incorporation into the alumina ceramic matrix was also shown to remain unchanged, suggesting that they are still capable of sustaining large elastic deformations (figures 15 and 16). Note that the values reported as the maximum sustainable loads corresponded to the partially ceramic-embedded MWCNTs (figure 9) and indeed are the lower bounds of their real load bearing ability (exploited when MWCNTs are fully embedded in the ceramic matrix) [10], i.e. the true load bearing ability of the MWCNTs in the ceramic matrix is expected to be even more superior than those reported; nevertheless, even these lower bound values are still considerably larger than those of near-perfect, straight, arc discharge-grown MWCNTs tested in vacuum [5, 6]. Such unique strengthening of the individual MWCNTs while embedded in the ceramic matrix could be attributed to the strong interwall shear resistance engineered inside the high-quality MWCNTs (load transfer and distribution inside the MWCNTs) due to the radial compressive stress applied from the surrounding ceramic matrix, which elastically compresses the MWCNTs in a radial direction (figure 14). Such radial elastic deformation is responsible for the formation of in-wall irregularities and thus the enhancement of the interwall shear resistance and maximum sustainable load. These highly energy dissipating failures of MWCNTs were characterized by a unique, unprecedented multiwall-type failure mode, as shown in figures 12 and 13, which suggests the contribution of inner walls in bearing the applied load and energy dissipation. Indeed, this was the first evidence that inner walls of the individual MWCNTs (which are generally unloaded and useless) could also contribute, in addition to the outermost wall, to the load bearing process and energy dissipation, i.e. the MWCNTs become dramatically stronger in the ceramic matrix by an interwall load-distribution mechanism [1, 9, 10]. In brief, these results confirmed that even MWCNTs with large deflections (opposite to what was proposed earlier [8]) are an exceptional reinforcement for ceramic-based materials and are superior to the expensive SWCNT bundles, though they require uniform dispersion within the matrix with intimate interfaces. The effect of such an unprecedented nanoscale energy dissipation mechanism on the macroscopic mechanical properties of the ceramic matrix composite, such as strength and toughness, will be reviewed in the next section.

## 5. Macroscopic mechanical properties

In this section, after providing a brief history on the mechanical properties of CNT–ceramic systems, we review the manifestation of the unprecedented in-MWCNT load transfer process characterized by the highly energy-dissipating multiwall-type failures (figures 12 and 13) [9, 10] in the macroscopic mechanical properties of the ‘clean’ MWCNT–Al_2_O_3_ composite system [1]. These composites are pore-free and are a structurally uniform system in which numerous MWCNTs at various concentrations are almost uniformly, individually and intimately dispersed on the grain boundaries of the Al_2_O_3_ ceramic in three dimensions with random orientations.

As briefly mentioned earlier in the introduction, the direct reinforcing ability of the CNTs in either SWCNTs or MWCNTs for ceramic-based materials have been seriously debated in the past decade, considering the disappointing results obtained mainly due to some well-known issues such as the agglomeration and non-uniform dispersion of CNTs, the poor interfacial compatibility leading to weak interfaces and negligible interfacial load transfer and the damage of CNTs during processing [15–43]. Although an intriguing 200% fracture toughness (*K*_IC_) improvement (9.7 MPa m^0.5^) was reported for a 10 vol % SWCNT–Al_2_O_3_ composite in 2003 [1], no convincing evidence of load transfer to and load bearing by the SWCNTs were provided. In addition, they used the Vickers indentation method, which is widely accepted as an indirect and unreliable method for the accurate measurement of the *K*_IC_ of ceramic-based materials and especially CNT–ceramic composites [18, 45]. Wang *et al* [18] reexamined the toughening phenomenon in a similar 10 vol % SWCNT–Al_2_O_3_ system and used instead the single-edge notched beam (SENB) method [80] for a direct and accurate *K*_IC_ measurement. Their study suggested that the claimed composite is as brittle as the dense monolithic Al_2_O_3_ ceramic (3.22 MPa m^0.5^) and 10 vol % graphite-Al_2_O_3_ composite (3.51 MPa m^0.5^) and revealed absolutely no toughening in that system (3.32 MPa m^0.5^). They argued that these composites are not tough at all but are contact-damage tolerant due to the presence of shear-deformable SWCNTs or graphite on the grain boundaries, which could effectively redistribute the intense stress field under the indenter, preventing the formation of classical radial cracks. Similar performances have been observed in the highly contact-damage tolerant, mica-based glass-ceramics, which are indeed brittle in nature, with the toughness ∼1.5 MPa m^0.5^ [81]. Indeed, the strengthening and toughening abilities of SWCNTs in a ceramic matrix are poor in our opinion, as they cannot be dispersed individually with direct interfaces with the matrix and exist mainly in bundled form [9, 18, 20, 21, 28, 31 and 32]. We should note that the possibility of energy dissipation by the individual SWCNTs of a SWCNT bundle during the crack bridging process seems very low simply because they are covered by neighboring easy-to-slide SWCNTs; thus, they cannot be tightly fixed by the matrix to allow their loading and contribution to the energy dissipation during the crack bridging process. Furthermore, we believe that the possible rupture and/or frictional pullout of the SWCNT bundles during the crack bridging process contributes marginally to the energy dissipation and the mechanical improvement.

**Figure 17.**
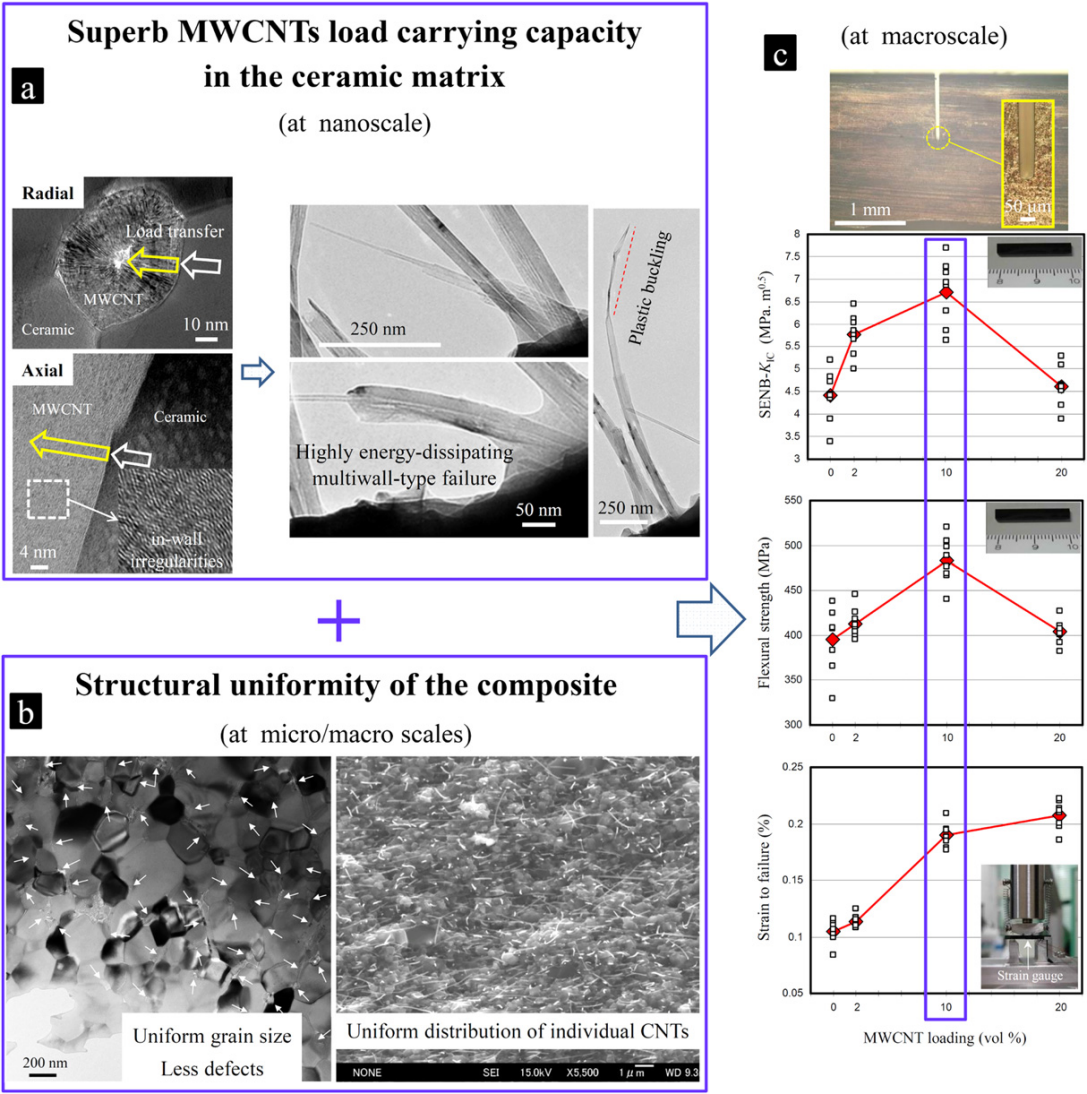
The combined effect of the (a) superb load carrying capacity of the individual MWCNTs in the ceramic matrix and the (b) structural uniformity of the ‘clean’ composite on the (c) macroscopic mechanical properties (SENB-*K*_IC_, flexural strength and strain tolerance): The mechanical properties in such a pore-free and structurally uniform ceramic matrix platform strongly depend on the MWCNT concentration that shows the best and unprecedented improvement at the rather large loading of 10 vol % [1, 9]. Reprinted from M Estili *et al* 2013 *Nanotechnology* **24** 155702 and M Estili and A Kawasaki 2010 *Adv. Mater*. **22** 607 (Copyright © 2010 WILEY-VCH Verlag GmbH & Co. KGaA, Weinheim).

**Figure 18.**
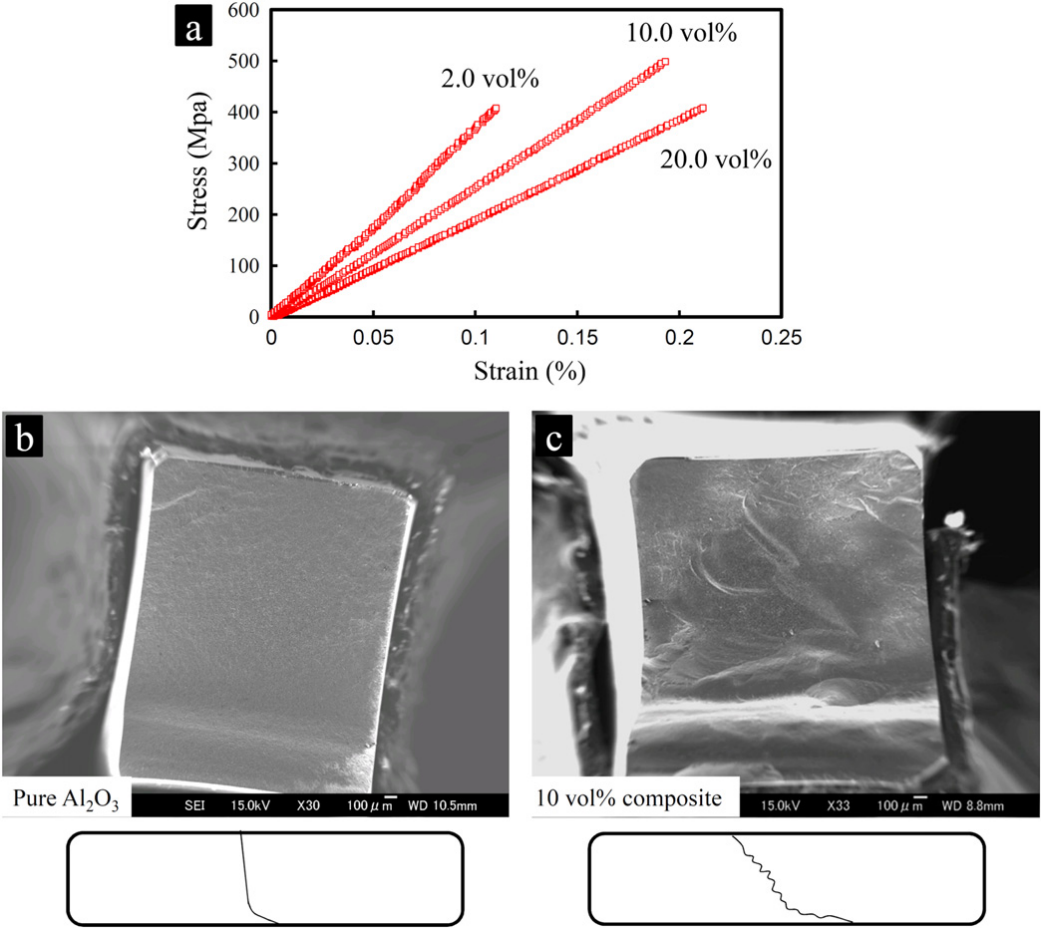
(a) Linear stress-strain responses of the 2.0, 10.0 and 20.0 vol % composites showing the increase of the strain tolerance with the MWCNT concentration. SEM images of the fracture surfaces of (b) the pure Al_2_O_3_ and (c) the 10 vol % composite: while the fracture surface is nearly flat and rather smooth in pure Al_2_O_3_, it is highly uneven and inclined in the 10 vol % composite, representing the larger extent of the crack deflection in the composite as evidence for the reported toughening [1]. Reprinted from M Estili *et al* 2013 *Nanotechnology* **24** 155702.

**Figure 19.**
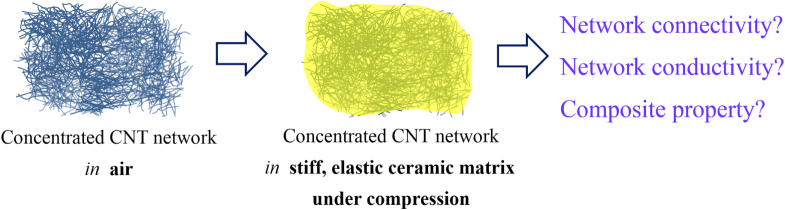
The concept of the CNT-concentrated ceramics [44].

**Figure 20.**
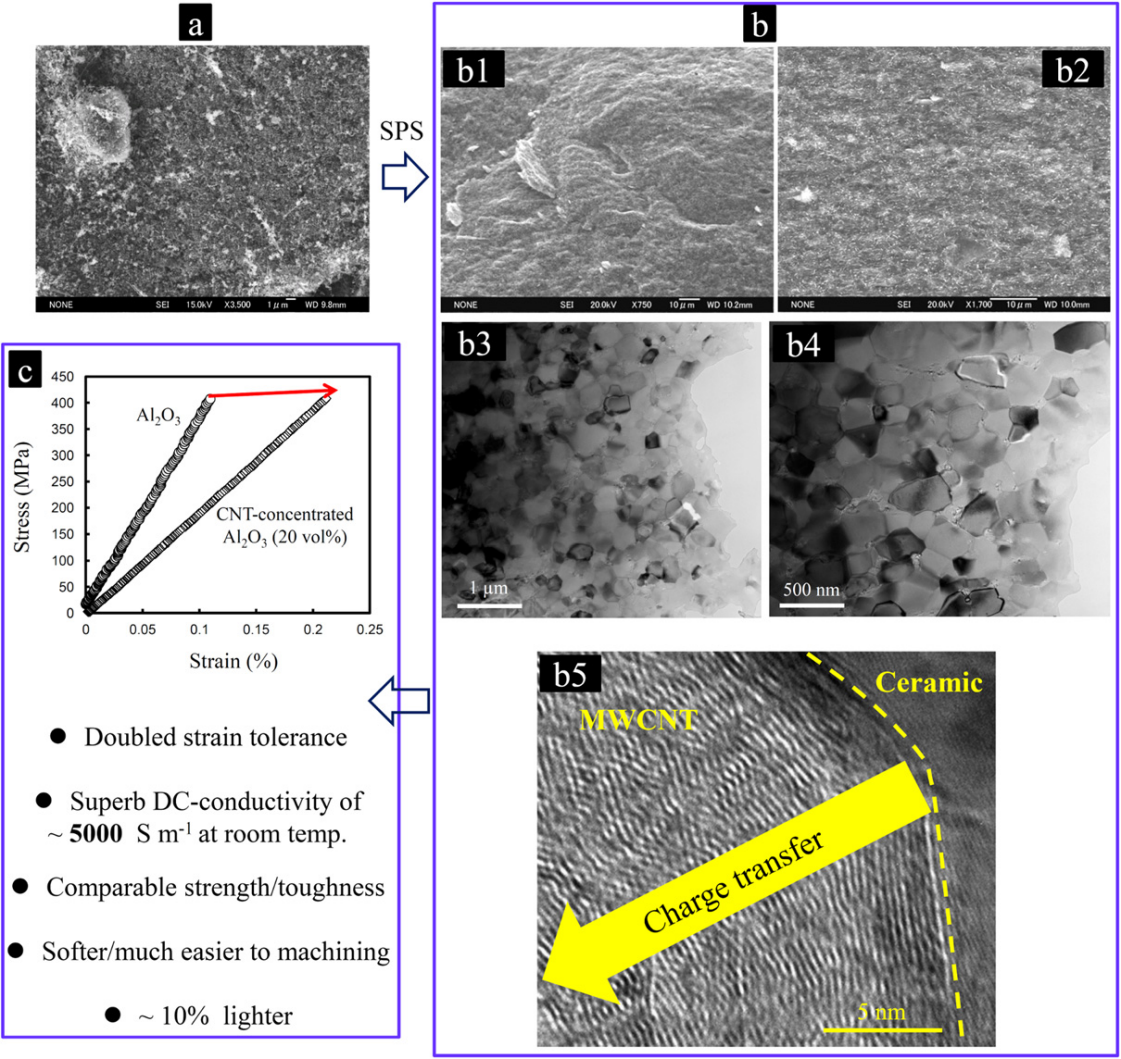
Microstructure and properties of the most CNT-concentrated ceramic bulk ever fabricated with nearly theoretical density in the MWCNT–Al_2_O_3_ composite system. (a) SEM image of the 20 vol % MWCNT–Al_2_O_3_ composite powder; (b) SEM (b1), (b2) and TEM (b3), (b4) images demonstrating the great structural uniformity of the sintered composite in terms of the individual CNTs dispersion and the matrix grain size distribution; and (b5) HRTEM image showing the in-wall irregularities in the MWCNT structure formed when embedded in the ceramic matrix, which are responsible for the charge transfer inside the MWCNT structure and for the increase of the conduction pathways, along with (c) the performance of the CNT-concentrated Al_2_O_3_ ceramic. The compressive-stressing ceramic matrix enhances the connectivity and thus the electrical conductivity of the concentrated CNT 3D-network by reducing the inter-tube junction resistance and increasing the walls’ connectivity inside the individual MWCNTs. On the other hand, the concentrated CNT network would greatly enhance the strain tolerance and machinability of the ceramic matrix without degrading the fracture toughness and strength [9, 44, 50]. Reprinted from M Estili and A Kawasaki 2010 *Adv. Mater*. **22** 607 (Copyright © 2010 WILEY-VCH Verlag GmbH & Co. KGaA, Weinheim), M Estili *et al* 2012 *Adv. Mater.* **24** 4322 (Copyright © 2012 WILEY-VCH Verlag GmbH Co. KGaA, Weinheim) and M Estili *et al* 2013 *J. Am. Ceram. Soc.* **96** 1904 (Â© 2013 The American Ceramic Society).

For the introduced ‘clean’ MWCNT–Al_2_O_3_ composite system, an unprecedented simultaneous enhancement was realized in the strain tolerance (81%, average 0.0019), the SENB-*K*_IC_ (52.2%, average 6.71 MPa m^0.5^) and the flexural strength (22%, average 483.19 MPa) in a rather high MWCNT concentration of 10.0 vol %, as summarized in figures 17 and 18 [1, 9, 10, 44]. Considering the lower weight of these composites (compared to monolithic Al_2_O_3_), the average improvements in the specific fracture toughness and flexural strength are then ∼62% and ∼30%, respectively. Note that the extent of toughening in such a ‘clean’ composite system (an average of 6.71 MPa m^0.5^ from eight reliable SENB-*K*_IC_ measurements: 7.68, 7.27, 7.15, 6.93, 6.86, 6.30, 5.87 and 5.64 MPa m^0.5^ [1]) is superior to those reported in the literature [15–43] while strongly supported by the convincing evidence of energy dissipation at the nanoscale as well as the structural uniformity at the micro- and macro-scales [9, 10, 44]. The considerable enhancement of the strain tolerance, in addition to the toughness and strength—all simultaneously—could even further enhance the mechanical reliability of the system by improving thermal shock resistance and the compatibility with secondary materials with the lower elastic moduli, such as metals, for the manufacture of complex metal-ceramic engineering components with reduced misfit stresses at joints [82–85]. Comparatively reduced but simultaneous improvements were also observed in composites with lower (2.0 vol %) and higher (20.0 vol %) concentrations, which demonstrates the vital effect of the CNTs concentration on the final macroscopic mechanical properties. In the 2.0 vol % composite (figure 6), the improvements in SENB-*K*_IC_, the flexural strength and the strain tolerance are limited to ∼31% (average 5.78 MPa m^0.5^), 4.5% (average 413.46 MPa) and 8.2% (average 0.0011). In the 10.0 vol % composite (figure 17), they are effectively increased to 52.2% (average 6.71 MPa m^0.5^), 22% (average 483.19 MPa) and 81% (average 0.0019), respectively. However, in the novel CNT-concentrated (20.0 vol %) composite (figures 6, 19 and 20), despite a nearly doubled strain tolerance, the simultaneous toughness and strength improvements are reduced to ∼4.7% (average 4.62 MPa m^0.5^) and 2% (average 403.72 MPa), respectively, which are even rather lower than the improvements achieved in the 2.0 vol % composite. In other words, an effective exploitation of the highly energy-dissipating multiwall-type failures of the crack-bridging MWCNTs (in-MWCNT load transfer) (figures 12, 13 and 14) in the final mechanical properties do indeed require a rather high but optimized concentration of MWCNTs, albeit in a pore-free and structurally uniform matrix platform. The optimized concentration is determined to be close to 10 vol % in such a ‘clean’ composite system [1]. Such an unprecedented performance using a high loading of functional acid-treated MWCNTs, namely toughening, strengthening, softening and lightening simultaneously and at this level, could have implications for many functional and structural applications.

The responsible mechanism could be explained as follows: looking at the stress-strain responses (figure 18), the elastic strain at a constant stress increases with the MWCNT concentration, which could be explained by the presence of MWCNTs on the grain boundaries of the alumina matrix. Note that the MWCNTs have largely different elastic moduli in axial and radial directions since they are soft and highly energy-absorbing in the latter [12–14]. The following deflection and bridging of the cracks of the ceramic matrix by those MWCNTs crossing the crack path (figure 10(a)), accompanied by their highly energy-dissipating, multiwall-type failures and plastic buckling (figures 12 and 13)—which occurred in a pore-free, structurally uniform matrix platform (figure 6)—could lead to the enhancements in the strength, toughness and thus the strain tolerance simultaneously (MWCNTs are stronger and tougher than the equivalent ceramic area they replace [9, 10]). Note that the grain refinement of the ceramic matrix due to the MWCNT addition could not be a determining reinforcing factor here because the grains are separated by the second-phase, radially soft CNTs with physical interfaces; for instance, the grain-size distribution in fully dense 10.0 vol % and 20.0 vol % composites is highly comparable, but the mechanical performance is largely different (figures 6, 17 and 20). It is our opinion that the grain-size effect should be discussed only for single-phase polycrystalline materials and not for the composites.

To explain the mechanical degradation upon excessively increasing the MWCNT concentration, the two different responses of the MWCNTs during crack propagation must be considered (see figure 10). These different responses arise because the interfaces in such ‘clean’ composites are weak in the normal direction (physical contact) but are very strong in shear mode due to the formation of strong mechanical interlocks between the matrix and MWCNTs’ surface defects (figure 11). The randomly oriented MWCNTs of our composite, depending on their orientation with respect to the crack path, could either bridge the crack (carry the applied load) or totally deflect the crack without carrying the load. The latter occurs if the MWCNTs lie on the crack surface (no crossing); then, they would easily detach from the ceramic matrix and totally deflect the crack around their interface—due to the weak physical contact with the matrix—and could return to their original relaxed figures after further crack opening. As mentioned in the previous section, the MWCNTs remain highly flexible even after incorporation into the Al_2_O_3_ matrix; if initially deflected over the rigid ceramic grains and laid partially on the crack surface, they would return to their original relaxed and straight figures as the crack opens (figures 15 and 16). These totally crack-deflecting MWCNTs do not carry the applied load and are even detrimental to the toughness, strength and strain tolerance simply because a covalent ceramic bonding is replaced with a weak physical CNT–ceramic one in this case. The former scenario, on the other hand, occurs if the MWCNTs cross the path of the propagating crack so that they first deflect the crack around their radial interface and then bridge the crack and carry the applied load through the strong interfacial shear resistance. Specifically, in the ‘clean’ composite described earlier, these bridging MWCNTs undergo a unique multiwall-type failure during the crack bridging process, which was experimentally found to be extremely energy-dissipating at the nanoscale (figures 12 and 13) [9, 10]. According to various crack-surface TEM-images similar to the one in figure 12, these useful crack-bridging MWCNTs (figure 10(a)) in this composite are nearly as populated as those deteriorating, totally crack-deflecting MWCNTs (figure 10(b)). With this short introduction in mind, the comparatively degraded mechanical performance of the MWCNT-concentered composite (20.0 vol %) could be due to the following deteriorating factors, which weaken the beneficial effect of the crack-bridging, highly energy-dissipating MWCNTs: (1) the existence of totally crack-deflecting (non-bridging) MWCNTs, which deteriorate the toughness, strength and strain tolerance (in this composite system, these MWCNTs seem to be as populated as the coexisting beneficial, crack-bridging MWCNTs); (2) the residual tension on the ceramic matrix [9, 86] (the more MWCNTs there are, the larger the ceramic area under residual tension); and (3) the possibility of formation of tiny, low-strength CNT aggregates (figure 17(b)), which increase with the concentration, though they are negligible in these composites according to many SEM and TEM images (these flaws, even in very small amounts/sizes, could be detrimental for a brittle matrix). For a given MWCNT concentration—in a pore-free, structurally uniform ceramic matrix platform—the final strength, toughness and strain tolerance are thus determined by a complex competition between the one beneficial factor and the above three deteriorating factors. In this ‘clean’ composite system, the best mechanical results are obtained at a rather high MWCNT concentration (10.0 vol %), which guarantees a considerably high strain tolerance (0.0019) that is very close to the maximum 0.0021 recorded in the 20.0 vol % composite; therefore, the structural reliability of this composite would be greatly enhanced [82–85]. However, the striking observation is that the beneficial effect of the highly energy-dissipating multiwall-type failures of the crack-bridging MWCNTs prevails over the as-mentioned deteriorating factors in a wide range of MWCNTs concentrations.

Such simultaneous strengthening, toughening and softening is unique, unprecedented and highly promising compared with the few reported simultaneous improvements realized only at low MWCNT concentrations (0.9 vol % [23], 3.0 vol % [24] and 4.0 vol % [22]) in which even a slight increase of the MWCNT concentration was shown to dramatically deteriorate both the toughness and strength. Ahmad *et al* reported simultaneous 94% (6.8 MPa m^0.5^) and 6.4% (380 MPa) enhancement in SENB-*K*_IC_ and strength for a hot-pressed 4.0 vol % MWCNT–Al_2_O_3_ composite. However, the *K*_IC_ improvement was reduced to 66% (5.8 MPa m^0.5^), followed by a 21% strength reduction (280 MPa) at a 10 vol % concentration [22]. Another work reported simultaneous SENB-*K*_IC_ and strength improvements of 25% (5.9 MPa m^0.5^) and 27% (689.6 MPa), respectively, with 0.9 vol % MWCNTs, albeit with a dramatic deterioration of mechanical properties even at a low 3.7 vol % MWCNTs concentration [23]. Similar improvements were also reported by Wei *et al* in a 3.0 vol % MWCNT–Al_2_O_3_ composite (79% (5.01 MPa m^0.5^) in SENB-*K*_IC_ and 13% (410 MPa) in strength), again with mechanical degradation above 3 vol % concentration [24]. In these studies [22–24], the dramatic deterioration of both the *K*_IC_ and strength upon increasing the MWCNT content could in principle bring into question the ability of their processing methods to effectively break the CNT agglomerates and achieve a uniform dispersion of individual CNTs within a pore-free and structurally uniform ceramic matrix, i.e. these methods are useful for fabricating composites with CNT concentrations below ∼4 vol %. Furthermore, the reported improvements were not supported by convincing evidence, confirming the load transfer to the CNTs and an effective load bearing and energy dissipation by the CNTs. For instance, strengthening (also toughening) was repeatedly attributed to the grain refinement as a side effect of the CNT addition, and the toughening was supported just by microstructural observations and explained by the classical mechanism proposed for the microscale, fiber/whisker-reinforced ceramic matrix composites. This old mechanism has been the debonding/crack deflection at the interface, followed by the crack bridging, which is then accompanied by the frictional pullout of fiber reinforcements [86–88]. However, the nanometer-scale size, remarkable flexibility and radial characteristics of CNTs [12–14], which are mostly located on the grain boundaries, make this classical mechanism inapplicable to the CNT–ceramic composite system mainly for two reasons: First, the nanoscale structure of CNTs could highly weaken the contributions of interfacial crack deflection and the frictional pullout during crack bridging (if any) due to dramatically smaller energy-dissipating zones [18, 86]. Second, in randomly oriented CNT–ceramic composites, crack deflection/debonding at the interface is only useful if it leads to crack bridging, followed by energy-dissipating processes such as frictional pullout or failure of CNTs (only for CNTs crossing the crack path) (figure 10(a)). On the other hand, frictional pullout of CNTs from the matrix (mostly claimed as the main toughening mechanism) cannot be confirmed just based on the observation of protruded (partially relaxed) CNTs on the fracture surface or of those seeming to bridge a crack. These features could simply occur either without friction (similar relaxed CNTs can also be seen on the fracture surface of porous CNT–ceramic composites where the pullout force is negligible) or just by the debonding and subsequent relaxation of the CNTs which lay on the crack surface (figure 10(b)) [10].

The simultaneous toughening, strengthening and softening realized in the ‘clean’ MWCNT–Al_2_O_3_ composite system for the first time is a unique combination of mechanical improvements impossible to achieve in traditional, microscale whisker/fiber-reinforced or other ceramic-based composites. Such an unprecedented performance using a high loading of functional MWCNTs could have implications for many functional and structural applications.

## 6. CNT-concentrated ceramics: a new concept toward the realization of multifunctional, strain tolerant ceramics

In this section, we review the novel concept, fabrication and properties of a unique CNT-concentrated ceramic, showing record-breaking electrical conductivity and doubled strain tolerance while maintaining the strength and toughness of the ceramic matrix even with marginal improvements. Breaking the conventional limit of CNT concentration in the matrix, these novel CNT-concentrated composites could be regarded as the new generation of CNT–ceramic composites, which were challenging to realize before, with various potential functional and structural applications.

The concept is simply the imagining of a concentrated three-dimensional (3D) network of CNTs in a ceramic environment instead of air (figure 19). In recent years, there have been growing interest and progress in the design and fabrication of CNT macrostructures (as film or bulk) for the effective utilization of its remarkable properties at the macroscale [89–99]. However, the realization of such concentrated CNT macrostructures in a protective, stiff/elastic ceramic matrix able to impose compression [9, 86] on the individual CNTs and their junctions could dramatically improve their network connectivity, charge transport properties and durability and perhaps lead to novel organic/inorganic composites with unprecedented multifunctional and even structural properties. Neither the concept nor its practical realization has been reported previously because of the great challenge of CNT agglomerations at such high loadings.

This great challenge was effectively addressed using a modified version of the scalable aqueous electrostatic hetero-coagulation approach reviewed earlier (figure 3) and could intimately embed the most concentrated (20.0 vol %) 3D macrostructure of MWCNTs ever inside of a *α*-Al_2_O_3_ matrix with pore-free and intimate interfaces (figure 20) [44]. The modification was simply the prolongation of sonicating the aqueous mixture (figure 3) until the aqueous MWCNT suspension was entirely added to the ceramic one. This strategy could effectively prevent the attraction and immobilization of a large number of ceramic nanoparticles by those MWCNTs added at the beginning of mixing process; therefore, more ceramic nanoparticles could become available to arrest the rest of the MWCNTs to be added; that is, more individual MWCNTs could then be incorporated within the ceramic powder with less possibility of secondary agglomeration. The collected composite powders consisting of numerous individual MWCNTs uniformly dispersed in three dimensions within the ceramic powder framework could then be transformed into a fully dense MWCNT-concentrated composite by the SPS technique [44, 53–60] (figure 6). This novel macrostructure showed an exceptional, room-temperature dc-electrical conductivity of nearly 5000 S m^−1^, approaching those of some single MWCNT nanostructures [100–102] and 3D hot-pressed, aligned [103] or highly compacted (∼90% dense) MWCNT bulks [104] but exceeding those of 3D sponge-like MWCNT bulks, either free-standing [95, 98, 99] or embedded, as in composites [35, 105, 106]. Such a remarkable charge transport property was demonstrated to originate from the internal compressive stresses formed by the ceramic grains, enabling the connection and exploitation of numerous, generally useless inner walls of individual MWCNTs serving as new conduction pathways and also the formation of intimate CNT/CNT local contacts with lower resistance (figures 14 and 20). Furthermore, such a CNT-concentrated network was shown to dramatically enhance the strain tolerance of the Al_2_O_3_ ceramic while improving the specific fracture toughness and strength by ∼17% and ∼14%, respectively [44]. The ability of CNTs to dramatically improve the strain tolerance of a ceramic material had not been reported in the literature previously. The mechanisms responsible for such mechanical reinforcement were described in detail in the previous section. Such highly improved strain tolerance could significantly enhance the reliability of the ceramic material, improving thermal shock resistance and the compatibility with lower-elastic-modulus materials (e.g. metals), which enable and stimulate the design and manufacture of complex metal-ceramic engineering components with largely reduced misfit stresses [82–85]. Note that, in general, at such high concentrations, degradation of toughness and strength is highly expected due to the ever increasing possibility of CNT agglomeration and pore formation; thereby, the unprecedented improvements realized strongly suggests the lowest degree of agglomeration in this ‘clean’ CNT-concentrated Al_2_O_3_.

This novel concept proposed by Estili *et al* could, in principle, stimulate multidisciplinary applied research on ‘CNT-concentrated ceramics,’ which are attractive for fields such as thermoelectric power generation, functionally graded ceramics, biomaterials, strain-tolerant and thermal-shock-resistant multifunctional ceramics, the design and manufacture of complex metal-ceramic engineering components, static-charge dissipation devices, electric-discharge manufacturing and many more, and it could open the door for the massive and sustainable utilization of low-cost, commercially available MWCNT nanostructures.

## 7. High-temperature mechanical properties

In the previous sections, we reviewed the important effect of the strong interfacial shear resistance on the effective load transfer to the MWCNTs and finally on the achievement of the unprecedented simultaneous strengthening, toughening and softening, but all at room temperature. It is scientifically and technologically important to examine the existence of such strong interfacial shear resistance at elevated temperatures and determine the resultant mechanical responses. In a CNT–ceramic system, there have been only a few reports on the high-temperature (HT) deformation behavior in compression and extrusion loading modes. The improvement of compressive creep resistance has been reported in 10 vol % SWCNT–Al_2_O_3_ [107–109] and 0.5–5.0 wt % MWCNT-ZrO_2_ composites [110]. These improvements were attributed to a grain boundary sliding (GBS) reduction as a consequence of CNT distribution in the grain boundary areas, which could lead to a grain boundary pinning effect and inhibit grain boundary diffusion due to a large grain boundary area covered by the CNTs. In the extrusion loading mode, which in general could produce higher strain rates compared to the compression mode, Peigney *et al* claimed that their CNTs (SWCNTs and double-walled CNTs) grown *in situ* within the metal-oxide ceramic matrix composite powders (Fe/Co-MgAl_2_O_4_ and Fe-Al_2_O_3_) could possibly enhance the extrusion speed (performed at 1500 °C under 43 MPa stress) through an easier GBS process (CNTs as a lubricating agent) and through inhibition of grain growth during the extrusion process [111]. In all of these reports, however, no information regarding the room-temperature interfacial shear resistance was provided. In this section, we review the recent research on the HT-mechanical response of the ‘clean’ MWCNT–Al_2_O_3_ composite system introduced earlier. The HT-compressive deformation behavior of a 20 vol % CNT-concentrated composite, which has a unique CNT-concentrated grain boundary structure, was investigated [50]. The HT-performance of this novel CNT-concentrated composite could be regarded as a reference for oxide systems in which the grain boundary areas are occupied with soft/elastic, highly energy-absorbing nanostructures.

**Figure 21.**
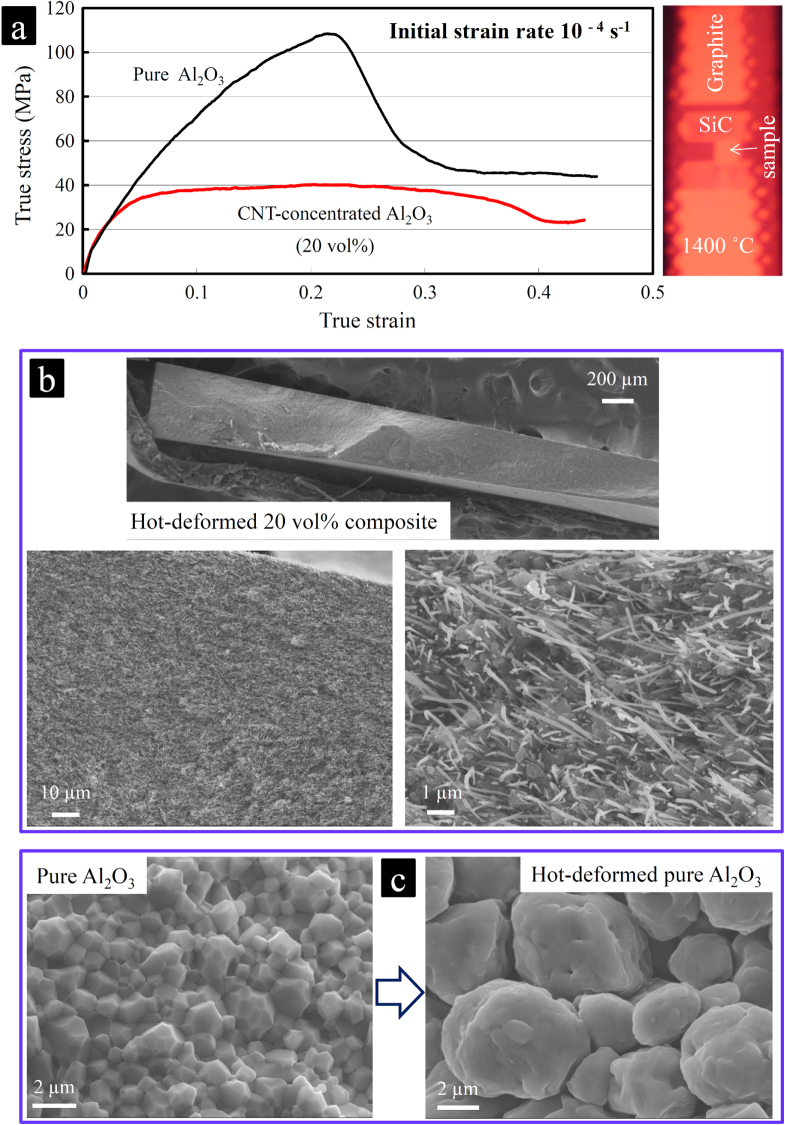
Perfect high-temperature plasticity realized in CNT-concentrated ceramic. (a) High-temperature compressive stress-strain responses of pure Al_2_O_3_ and the CNT-concentrated Al_2_O_3_ composite (20 vol %) performed in a highly pure argon atmosphere; and (b), (c) SEM images of their fracture surfaces. The composite shows a perfect plastic deformation behavior at a small ∼30 MPa flow stress, contrary to the typical strain hardening response of the fine-grain pure Al_2_O_3_ ceramic leading to its extensive cavitation (c). According to the SEM images of the hot-deformed composite (b), the MWCNTs uniformly distributed in the GB area ideally withstand not only the high temperature but also the shear/compressive forces and strongly preserve the nanostructure of the ceramic matrix by preventing the dynamic grain growth, even during ∼44% compressive deformation [50]. Reprinted from M Estili *et al* 2013 *J. Am. Ceram. Soc*. **96** 1904 (Â© 2013 The American Ceramic Society).

**Figure 22.**
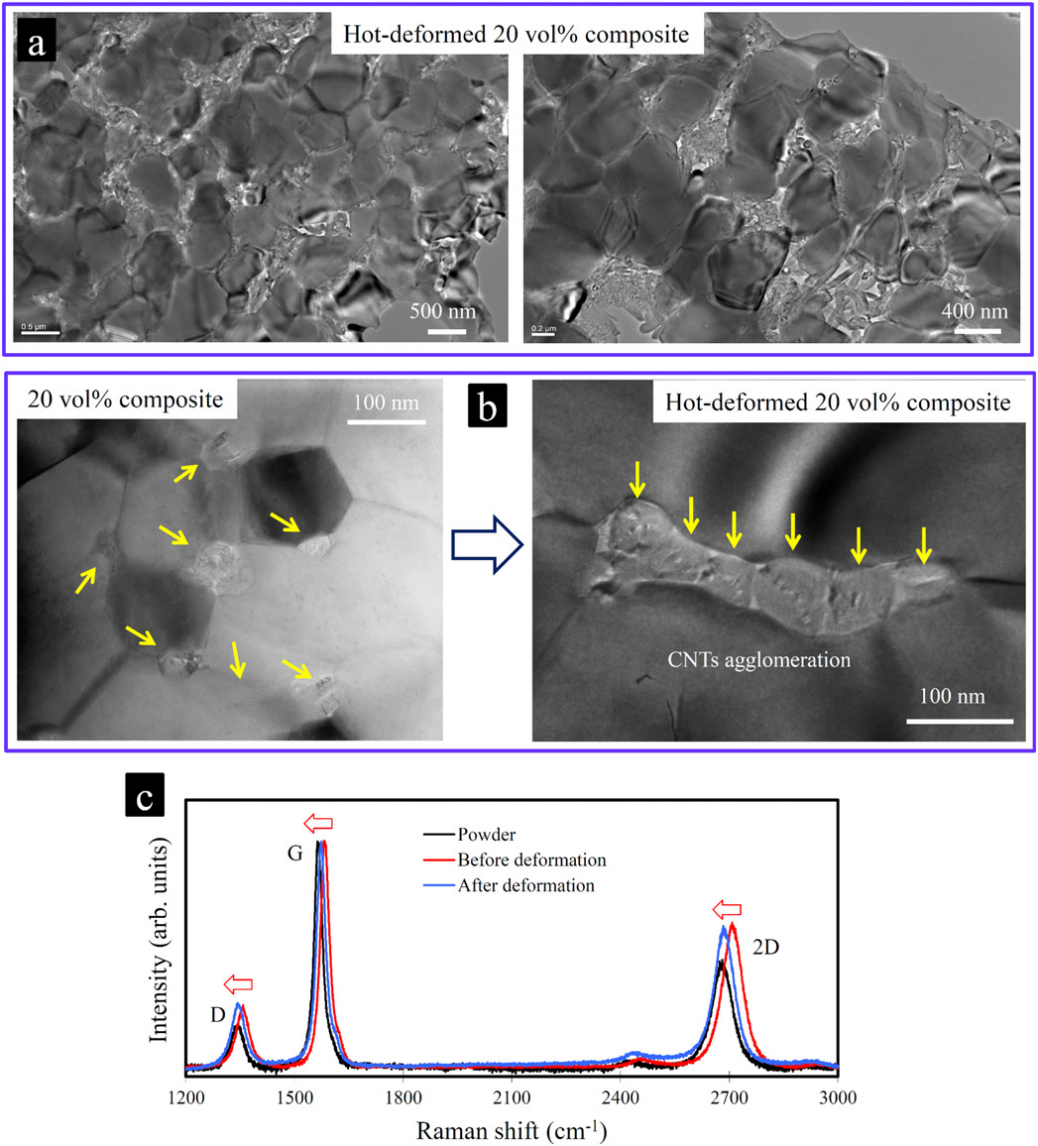
Microstructure of the hot-deformed CNT-concentrated ceramic. (a), (b) TEM images of the hot-deformed CNT-concentrated Al_2_O_3_ composite (20 vol %) showing slight grain growth and MWCNTs agglomeration. Such slight agglomeration could be avoided by decreasing the CNT concentration of the composite (e.g. 10 vol %) for the practical application. (c) The Raman spectra of the CNT-concentrated composite powder, sintered bulk and the hot-deformed bulk composite showing almost no damage to the MWCNTs and a considerable release of the residual stresses during the deformation at high temperature [50]. Reprinted from M Estili *et al* 2013 *J. Am. Ceram. Soc*. **96** 1904 (Â© 2013 The American Ceramic Society).

For the HT-compressive deformation experiments, the cut specimens (2.5 mm × 3.0 mm × 5.0 mm) were deformed perpendicular to the SPS direction at the initial strain rate of 10^−4^ s^−1^ and at a constant temperature of 1400 °C in a highly pure argon atmosphere under uniaxial compression. Three experiments were performed, and the stress-strain responses were highly comparable. The temperature of the furnace with a tungsten heating element was gradually raised to 1400 °C (at a rate of about 15 °C min^−1^) and maintained there for 10 min prior to the loading. True stresses were calculated using the true cross-sectional area by assuming a constant volume of specimens during the deformation. Looking at the stress-strain responses (figure 21), the CNT-concentrated composite, despite the strong room-temperature interfacial shear resistance, is characterized by a perfect plastic behavior at the rather high initial strain rate of 10^−4^ s^−1^, with a flow stress as low as 30 MPa, which is smaller than that observed in the 10 vol % ZrO_2_-Al_2_O_3_ matrix composite (∼40 MPa) in a similar deformation condition [112]. In contrast, the monolithic Al_2_O_3_ shows a typical strain hardening behavior [112–114] due to the instability of its microstructure and dynamic grain growth during the deformation [115–121]. The large extent of the cavitation and eventual cracking leads to the sharp stress-drop from ∼108 MPa (at ∼ 22% deformation) [112] because the thermally activated processes, such as GBS, cannot be further fully accommodated by the lattice/grain boundary diffusion for the large micrometer-scale grains, which leads to stress rising and cavitation. Looking at the fracture surface images of the hot-deformed specimens at 1400 °C with ∼44% deformation (60 min) (figure 21), the composite has a very fine, seemingly pore-free and uniform structure similar to the one before the deformation, while monolithic Al_2_O_3_ suffered from an extensive grain growth and pore formation. Furthermore, the 1D structure and crystallinity of MWCNTs (according to the Raman spectra in figure 22) are preserved, and their distribution within the matrix still looks uniform despite the large plastic flow. However, according to the TEM images (figure 22), the matrix grain size is slightly increased (from 150–350 nm to 0.5–1 *μ*m), which is indeed expected either after ∼44% deformation or by a deformation temperature higher than the sintering temperature (1300 °C), which could produce grain growth, as reported in the literature. In addition, some tiny, nanoscale CNT aggregates are formed in the GB areas, although no porosity could be observed between the dislocation-free and equiaxed matrix grains. The formation of such aggregates must be avoided in general because it could eventually lead to cavitation and cracking due to disruption of the uniformity of the CNTs dispersion within the matrix, leading to the formation of a CNT-free area and subsequent dynamic grain growth. Such cavitation in the CNT-concentrated composite is indeed expected and could have started from ∼22% deformation, and it is characterized by a slow stress-drop (figure 21). For the practical application, it is suggested that the MWCNT concentration be decreased (e.g. 10 vol %) to avoid such aggregation. It is interesting to compare the deformation behavior of this composite with that of the SWCNT–Al_2_O_3_ one reported earlier [112–114]. In the SWCNT–Al_2_O_3_ composite, the SWCNTs act as a rigid phase (unlike the radially soft/elastic, highly energy-absorbing MWCNTs), completely inhibiting the grain boundary mobility or sliding (grain pinning) and suppressing the grain boundary diffusion; deformation is produced by dislocation sliding. This could explain the comparatively lower strain rates achieved in the SWCNT composite, which makes it a creep-resistant one (extrapolation to 1400 °C results in the strain rates of ∼10^−5^ and 4 × 10^−6^ s^−1^ for pristine and acid-treated SWCNT–Al_2_O_3_ composites (at 50 MPa), which could become even lower at ∼30 MPa (flow stress of our MWCNT–Al_2_O_3_ composite). It is clear that the CNT type could greatly influence the HT-deformation behavior of the Al_2_O_3_ matrix.

In brief, the MWCNTs withstood not only the high temperature but also the shear/compressive forces involved and effectively preserved the structure of the ceramic matrix by preventing the dynamic grain growth, even during large compressive deformation performed at the high temperature of 1400 °C and a rather high strain rate of 10^−4^ s ^−1^. This is regarded as the first prerequisite of the perfect plastic behavior [50, 112–121]. In general, the mechanism of the large plastic deformation could not be exactly determined by mechanical tests at only one temperature and strain rate; however, in our opinion, the formation of the mentioned MWCNT aggregates in the grain boundary area and the observation of dislocation-free matrix grains with no interfacial porosity and almost a similar shape factor to that before the deformation could occur only if the GBS mechanism, accommodated by the grain boundary/lattice diffusion, had been effective and facilitated during the deformation. However, an important prerequisite for the GBS process to be facilitated would be a weak interfacial shear resistance at 1400 °C. A direct evaluation of the interfacial shear resistance at 1400 °C is technically very challenging, however, the disappearance of peak-shifts in the Raman spectra (figure 22) after the deformation could indirectly support the weakening of the interfacial shear resistance at 1400 °C. In the ‘clean’ MWCNT-concentrated composite system, the collective Raman peak-shifts to the higher frequencies after full densification was previously proved to be due to the generation of internal misfit compressive stresses, which elastically compress the MWCNTs in a radial direction (figures 7 and 14) [9, 86]. Therefore, their disappearance would suggest a considerable release of the residual stresses during the deformation at 1400 °C. Such a stress relaxation could weaken the interlocking resistance (shear resistance) at 1400 °C between the matrix grains and the nanoscale surface defects of the MWCNTs. Indeed, the presence of a large amount of radially soft/elastic, highly energy-absorbing MWCNTs in the grain boundary and specially multiple junction areas, with no covalent bonding to the matrix and a potentially weak interfacial shear resistance at 1400 °C, could greatly facilitate the GBS process and simultaneously prevent the dynamic grain growth [112–121], as evidenced by the formation of MWCNT aggregates in the grain boundary area, the equiaxed grains and the intra-grain dislocation-free structure of the deformed composite (figure 22). The MWCNTs seem not to hinder the accommodation of the GBS process by the lattice/grain boundary diffusion mainly due to their uniform dispersion and nanoscale radial dimension, though they significantly suppress the dynamic grain growth. The perfect plastic deformation behavior realized in this system is attractive for the ceramic forming industry and could be used as a reference for the HT-deformation studies on the oxide systems in which the grain boundary areas are occupied with soft/elastic, highly energy-absorbing nanostructures.

## 8. Future directions

Thanks to the scalable processing method, which pushes the conventional boundaries of CNT concentration in the ceramic matrix and enables the fabrication of CNT-concentrated ceramics with unprecedented mechanical and charge transport properties (as demonstrated in the MWCNT–Al_2_O_3_ matrix composite system), the main directions for future research could be: (1) CNT-concentrated ceramics and (2) CNT–ceramic FGMs. These CNT-concentrated and functionally graded composite materials would surely offer multifunctional properties for challenging functional structural applications, from bio to energy.
